# Association of *BUD13*-*ZNF259*-*APOA5*-*APOA1*-*SIK3* cluster polymorphism in 11q23.3 and structure of APOA5 with increased plasma triglyceride levels in a Korean population

**DOI:** 10.1038/s41598-019-44699-x

**Published:** 2019-06-05

**Authors:** Han-Kyul Kim, Muhammad Ayaz Anwar, Sangdun Choi

**Affiliations:** Department of Molecular Science and Technology, Ajou University, Suwon, 16499 Korea

**Keywords:** Data mining, Genome informatics

## Abstract

In this association study on chromosome 11, the data from 12,537 Korean individuals within the Health Examinee (HEXA) and the Korea Association Resource (KARE) projects were analysed to identify genetic loci correlating with increased and decreased plasma triglyceride (TG) levels. We identified a locus in chromosomal region 11q23.3 that harbours genes *BUD13*, *ZNF259*, *APOA5*, *APOA1*, and *SIK3*, which may be associated with plasma TG levels. In this locus, 13 relevant single-nucleotide polymorphisms (SNPs) were found: rs184616707, rs118175510, rs60954647, rs79408961, and rs180373 (near *BUD13*); rs11604424 (in *ZNF259*); rs2075291, rs651821, and rs7123666 (in or near *APOA5*); rs525028 (near *APOA1*), and rs645258, rs10160754, and rs142395187 (in or near *SIK3*). All 13 SNPs satisfied the genome-wide significance level (P < 5.0 × 10^−8^) in both meta-analysis and conditional analysis. Haplotype analysis of six SNPs (rs79408961, rs180373, rs2075291, rs651821, rs525028, and rs10160754) that were selected based on the β coefficient and conditional *P* values, revealed nine common haplotypes (with frequency 0.02–0.34) associated with both increased and reduced TG levels. Furthermore, to shed light on possible structural implications, we modelled and simulated the G185C variant of APOA5 (corresponding to rs2075291), which showed the strongest association. Molecular dynamics simulation results showed that this polymorphic variant of APOA5 has a different hydrogen bond network, increased average distance between chains, and an ability to form distinct clusters. Owing to the orientation of cysteine, the possibility of disulphide bond formation with other proteins is evident. In summary, our association and modelling analyses provided evidence that genetic variations in chromosomal region 11q23.3 are associated with elevated TG levels.

## Introduction

Plasma triglyceride (TG) levels play a significant role in metabolic disorders, including insulin resistance, hyperglycaemia, hypertension, and obesity^1,2^. Plasma TG levels are influenced by environmental as well as heritable factors^3^; the latter are associated with an increased risk of cardiovascular diseases^4^. Through a series of processes, TGs are usually secreted from the liver as very low-density lipoprotein (VLDL) particles, which are composed of TG, cholesterol, phosphatidylcholine, and apolipoprotein^5^. Approximately 50% of VLDL particles are transferred into the cell through receptor-mediated endocytosis via interactions between apolipoprotein E and low-density lipoprotein receptors^6^. In addition, apolipoprotein A5 (APOA5), which belongs to the apolipoprotein family, plays a pivotal role in TG level regulation and typically reduces VLDL production^7^. Van der Vliet *et al*. have demonstrated that mice overexpressing APOA5 show a 70% reduction in the TG level as compared with wild-type (WT) mice^8^. This reduction in plasma TG concentration is attributed to changes in VLDL turnover^9^. In contrast, *APOA5* knockout mice have a plasma TG level four-fold higher than that of control mice^10^. This observation suggests that APOA5 affects plasma TG levels, and that dysfunction of *APOA5* may be a risk factor of hypertriglyceridaemia^7^.

Some genome-wide association studies (GWASs) have revealed that genetic variants can significantly increase plasma TG levels, and *APOA5* and lipoprotein lipase are well known TG-related genes^11–13^. Furthermore, in the Chinese Han population, the rs651821 polymorphism in *APOA5* is strongly associated with plasma TG levels^14^; and in 2015, 41 additional loci in Europeans were associated with TG levels through GWASs^13,15^.

Various GWAS findings have indicated that the *BUD13*-*ZNF259*-*APOA5*-*APOA1*-*SIK3* gene cluster in the 11q23.3 chromosomal region is strongly involved in TG metabolism^16–18^. Therefore, in this study, we investigated the *BUD13*-*ZNF259*-*APOA5*-*APOA1*-*SIK3* gene cluster in a Korean population, and found 13 SNPs, which span 465 kbp. In the 11q23.3 region, we then determined the effects of haplotype variants. To this end, we obtained access to the data on Korean cohorts, the HEXA (Health Examinee) study (discovery study) and KARE (Korea Association Resource) study (replication study), and performed statistical analysis. We found that the G185C variant of APOA5 has the strongest association with TG levels in this combined cohort of Korean population. Furthermore, to supplement our experimental data, various computational approaches were used including molecular modelling and molecular dynamics (MD) simulations. Accordingly, we created a homology model for G185C in APOA5 (rs2075291), the only SNP with the highest probability to be expressed and to cause the G185C substitution in APOA5, whereas the other SNPs are located in non-protein-coding regions. Finally, the models were subjected to MD simulations to lend support to our genetic findings.

## Results

The overall study design is presented in Fig. S1. The general characteristics of the study participants are provided in Table 1. The mean TG levels were higher in KARE participants (162.9 mg/dl) than in HEXA participants (123.3 mg/dl).

**Table 1:** Baseline characteristics in the discovery (HEXA) study and replication (KARE) study.

Group	Discovery (HEXA)	Replication (KARE)	P value
No. of subjects	3,689	8,834	
Males, n (%)	1,646 (44.6)	4,176 (47.3)	0.006
Age, y^α^	53.2 ± 8.3	52.2 ± 8.9	<0.001
Body mass index, kg/m^2^	24.0 ± 2.9	24.6 ± 2.9	<0.001
TG, mg/dl	123.3 ± 90.6	162.9 ± 105.7	<0.001
Fasting glucose, mg/dl	94.1 ± 24.6	87.7 ± 21.9	<0.001
TCHL, mg/dl	197.5 ± 35.0	191.6 ± 36.0	<0.001

^α^Data are shown as mean ± standard deviation.

TG, Triglyceride; TCHL, Total cholesterol.

To find gene associations with plasma TG levels among these Korean participants, the data from these studies were subjected to various analyses. As a result, a locus (*BUD13*-*ZNF259*-*APOA5*-*APOA1*-*SIK3*) in chromosomal region 11q23.3 was found to be associated with TG levels. When we analysed chromosome 11 using a mixed-linear model, a total of 426 SNPs satisfied the genome-wide significance level (*P* < 5.0 × 10^−8^) after meta-analysis with the replication study, and all these 426 SNPs are located in the 11q23.3 region (Table S2). Next, we performed conditional analysis on these 426 SNPs and as a result only 13 SNPs remained (*P* < 5.0 × 10^−8^). Out of these 13 SNPs in the *BUD13*-*ZNF259*-*APOA5*-*APOA1*-*SIK3* gene cluster, five SNPs were near *BUD13*; one SNP was in *ZNF259*; three SNPs were in or near *APOA5*; one SNP was near *APOA1*; and three SNPs were in or near *SIK3*.rs651821 was found to have the highest statistical significance (combined *P* = 4.91 × 10^−100^). Relevant details for these SNPs, including neighbouring genes, SNP ID, chromosomal position, minor allele frequency (MAF), the effect allele, and effect sizes are provided in Table 2.

**Table 2:** A summary of SNPs related to significant variants in the discovery (HEXA) and replication (KARE) studies.

Nearby Gene^a^	SNP ID	Class	Position^b^	Effect allele^c^	Discovery (HEXA) study		Trend *P* value^d^			Conditional *P* value	Beta of combination
					MAF	Beta	HEXA (*n* = 3,689)	Replication study (KARE) (*n* = 8,834)	Combined (*n* = 12,535)		
BUD13	rs184616707	NearGene-3	116510558	G	0.02	35.52	5.49E-05	1.85E-05	4.13E-09	2.47E-13	34.11
BUD13	rs118175510	NearGene-3	116532548	C	0.10	9.10	9.91E-03	6.74E-08	5.22E-09	2.50E-10	12.71
BUD13	rs60954647	NearGene-3	116566933	T	0.47	−7.49	3.79E-04	4.29E-08	7.34E-11	5.13E-143	−8.28
BUD13	rs79408961	NearGene-3	116588593	T	0.07	13.12	9.67E-04	2.05E-16	1.01E-17	1.06E-144	20.23
BUD13	rs180373	NearGene-3	116591553	A	0.10	−9.12	8.53E-03	5.09E-09	3.28E-10	2.53E-68	−12.65
ZNF259	rs11604424	Intron	116651115	C	0.49	7.44	2.80E-04	2.53E-11	7.13E-14	1.56E-178	9.52
APOA5	rs2075291	Missense	116661392	A	0.07	37.11	6.46E-21	8.30E-48	9.60E-67	7.91E-109	40.53
APOA5	rs651821	Intron	116662579	C	0.29	23.46	9.61E-26	1.98E-78	4.91E-100	8.57E-213	29.25
APOA5	rs7123666	NearGene-5	116667083	A	0.14	12.80	2.17E-05	3.60E-15	9.29E-19	4.35E-14	15.99
APOA1	rs525028	NearGene-3	116705516	A	0.25	−8.51	4.00E-04	7.64E-07	1.26E-09	5.29E-90	−9.11
SIK3	rs645258	Intron	116801201	A	0.29	4.87	2.96E-02	1.36E-10	2.08E-10	2.82E-14	8.96
SIK3	rs10160754	Intron	116858994	C	0.14	−9.60	1.47E-03	5.64E-08	4.38E-10	1.52E-78	−11.54
SIK3	rs142395187	NearGene-5	116976009	G	0.12	−13.10	1.07E-04	9.69E-09	5.18E-12	2.52E-13	−14.52

^a^Nearby genes are defined as the closest genes to the SNP within signal boundary or the closest genes within a 200-kb window.

^b^Chromosomal positions are based on the 1000 Genomes Project’s haplotype phase I in NCBI build 37 (hg19).

^c^The minor allele is the effect allele.

^d^The P value for the effects of genotypes on plasma TG levels were assessed via a mixed linear model with adjustment for age, sex, and body–mass index (BMI).

**Figure 1:**
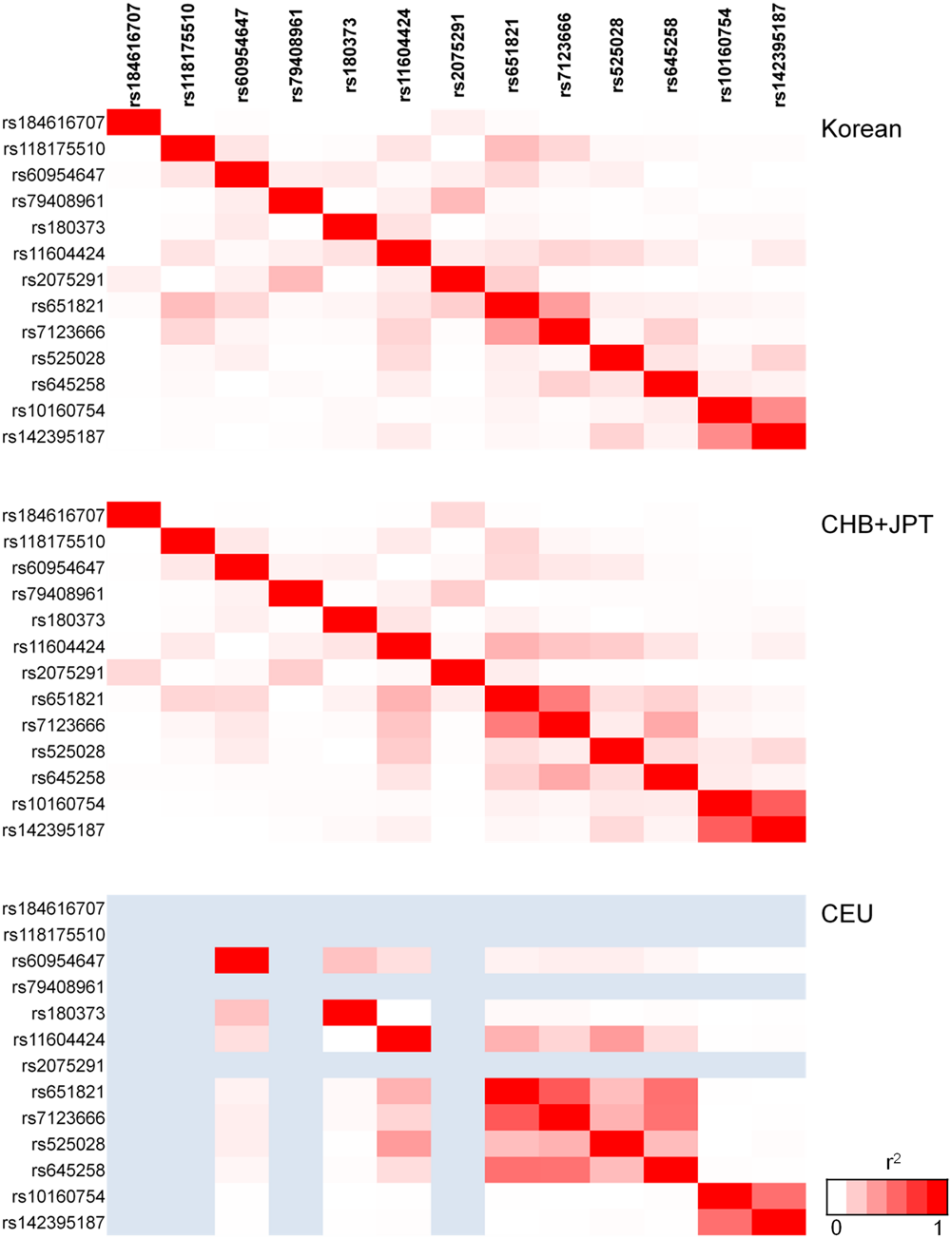
LDlink matrix patterns among the 13 SNP targets in Korean, Chinese, Japanese, and European populations. Genes present in chromosomal region 11q23.3 include *BUD13*, *ZNF259*, *APOA5*, *APOA1*, and *SIK3*. Pairwise LD values between the SNPs are described by white-red and blue shading; r^2^ = 0 is represented by the white colour, r^2^ = 1 is represented by red, and blue denotes intermediate LD.

The linkage disequilibrium (LD) matrix pattern among the 13 markers in the 11q23.3 region is presented in Fig. 1. LD profiles of the 13 markers found in the combined Korean population were comparable with those of Chinese and Japanese (CHB/JPT) populations but differed from those of Europeans (CEU). The rest of the SNPs were in weak LD (r^2^ < 0.30), whereas rs10160754 and rs142395187 (r^2^ = 0.46, in the combined Korean population) were the exceptions.

Among the 13 SNPs, only eight SNPs satisfied the conditional P value (*P* < 2.5 × 10^−68^). From these eight SNPs, we selected six SNPs: three with the highest and three with the lowest β coefficients. Next, to test whether the six selected SNPs have any association with plasma TG levels, we conducted a haplotype analysis on the selected SNPs; haplotypes with <1% frequency were excluded from further analyses. Nine haplotypes were obtained, and the details are shown in Table 3. When the most common CCCTGA haplotype was used as a reference, the other three haplotypes, CCACGA (β = 44.25, *P* < 10^−5^), TCACGA (β = 40.10, *P* < 10^−5^), and CCCCGA (β = 17.82, *P* < 10^−5^) showed a significant correlation, whereas one haplotype was only marginally associated with TG levels. In contrast, the other four haplotypes showed a weak association with lower TG levels in the combined Korean population.

**Table 3:** Association of the haplotypes involving the six SNPs in the Korean populations.

rs79408961 (*BUD13*)	rs180373 (*BUD13*)	rs2075291 (*APOA5*)	rs651821 (*APOA5*)	rs525028 (*APOA1*)	rs10160754 (*SIK3*)	Haplotype Frequencies	β coefficient	*P*
C	C	A	C	G	A	0.02	44.25	<1.00E-05
T	C	A	C	G	A	0.04	40.10	<1.00E-05
C	C	C	C	G	A	0.20	17.82	<1.00E-05
C	C	C	T	A	A	0.15	0.34	2.09E-02
C	C	C	T	G	A	0.34	Reference	6.00E-05
C	C	C	T	G	C	0.05	−2.88	1.86E-02
C	A	C	T	G	A	0.06	−5.11	1.34E-02
C	C	C	T	A	C	0.04	−7.05	6.01E-03
T	C	C	T	A	C	0.02	−8.27	3.40E-02

**Figure 2:**
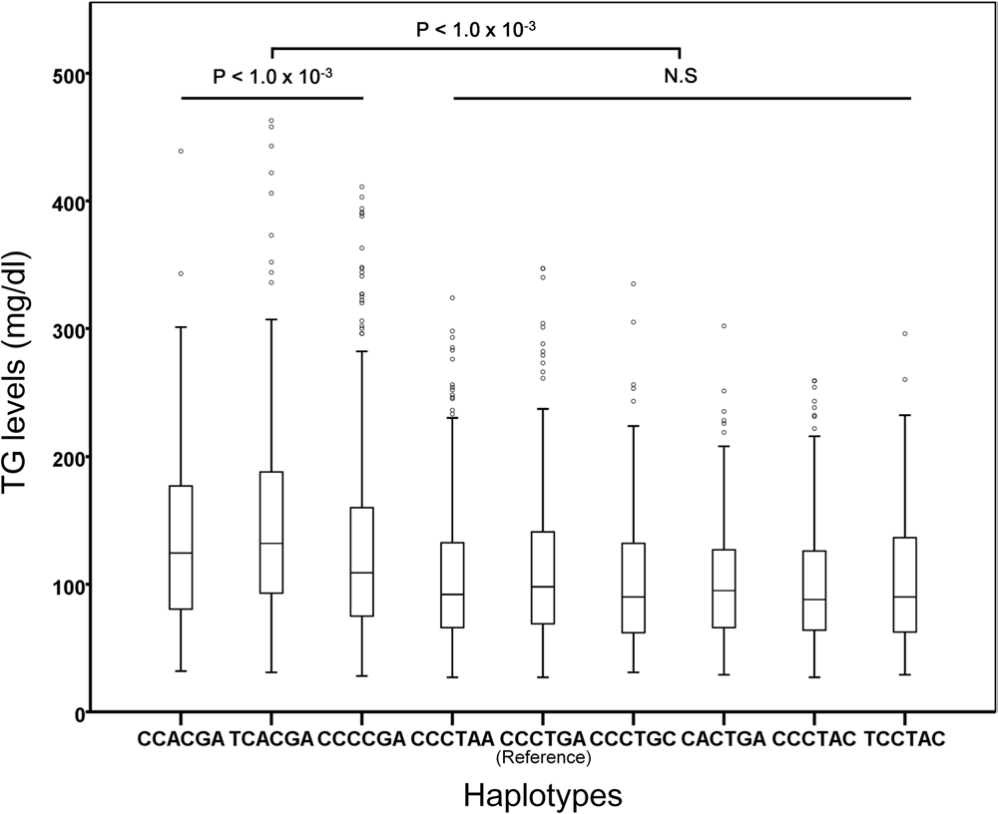
Plasma TG levels in people with haplotypes containing the six SNPs. A box plot of plasma TG levels in people with the identified haplotypes. Outliers are indicated by small circles, far outliers are not shown in this figure.

Next, we determined whether allelic dosage of risk haplotypes containing the six SNPs contributed to quantitative diversity in plasma TG levels of the 3,689 individuals from the HEXA study. As shown in Fig. 2, plasma TG levels were evaluated according to haplotypes. Plasma TG levels were significantly higher in people with haplotype CCACGA, TCACGA, or CCCCGA (*P* < 10^−3^). The other five haplotypes showed no correlation with plasma TG levels as compared with those of controls.

Among the polymorphisms, the only SNP that can cause an amino acid substitution in a protein is rs2075291 (in APOA5). This SNP can result in G185C substitution, where the addition of cysteine can potentially provide an opportunity for formation of new disulphide bond with other proteins. In addition to the possibility of a disulphide bond, this mutation can decrease the protein’s flexibility and can rearrange its three-dimensional (3D) structure resulting in an altered conformational ensemble. The altered state of protein conformation can significantly change its functional characteristics. To test this hypothesis, a homology model was created, and docking was performed for dimerisation of APOA5. The resultant dimer showed that C185 faces outward, and the relative positions of neighbouring C185s are too far from each other for possible disulphide bond formation. Similarly, C185 was unable to form a disulphide bond with C227 (Fig. S2). This result suggested that the probability of disulphide bond formation between these monomers is low, unless they slide along in the opposite directions to ensure the proper distance needed for bond formation. Nevertheless, due to the easily accessible -SH group in this model, it is highly likely that a disulphide bond will form between a dimer of APOA5 and other interacting proteins, such as kininogen-1 and fibronectin^19^. This additional bond formation may result in a loss of function or an unusual multimeric complex. Moreover, the individual monomers in this APOA5 dimer model are antiparallel, and thus can snugly fit into each other to support the dimer formation.

### Structural modelling of polymorphic APOA5

**Figure 3:**
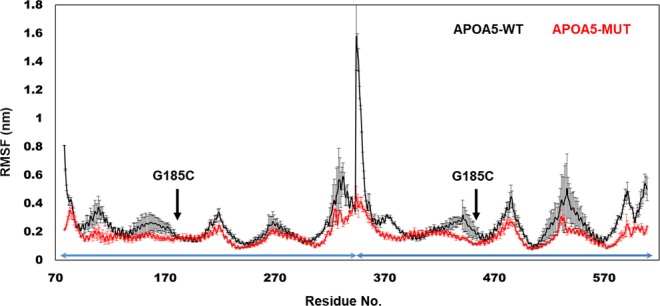
The residual fluctuation of APOA5 complexes. RMSF was calculated to monitor the amino acid mobility. The WT complex (black curve) manifested greater fluctuation along its length as compared to the mutant (red). RMSF is the average of last 10 ns of two independent simulations; a standard deviation in the respective colours is presented too. The monomeric part of this homodimeric complex is indicated by an arrow along the X-axis.

To verify these findings and to evaluate the structural features APOA5, MD simulations were performed on APOA5-WT and APOA5-MUT (G185C). These dimeric molecules either appeared to be stable when analysed using root mean-squared deviation (RMSD) and radius of gyration (Rg) or acquired stability later during simulations (Fig. S3A,B). These systems showed more variability in the initial phase of MD simulations, and variability was also evident within the same system. With time, however, the systems reached an equilibrium and converged to similar values. Readers should take into account the chaotic nature of MD simulations where even the same system can evolve to different conformation. The per residue fluctuation data also provided interesting insights: APOA5-WT showed greater fluctuation than APOA5-MUT did (Fig. 3). The results indicated that APOA5-WT is more flexible than the polymorphic form of APOA5, and such structural confinement may be due to the presence of C185. This finding is expected because glycine is a flexible residue with hydrogen as a side chain, protein regions with glycine usually form loop structures due to its flexibility. When glycine is replaced with cysteine, protein region becomes more rigid^20^.

Secondary structures of the protein remained largely intact throughout the simulation when analysed through dictionary of secondary structure of proteins. No substantial changes were observed in any secondary structure classes; however, minor alterations were expected (Fig. S4).

**Figure 4:**
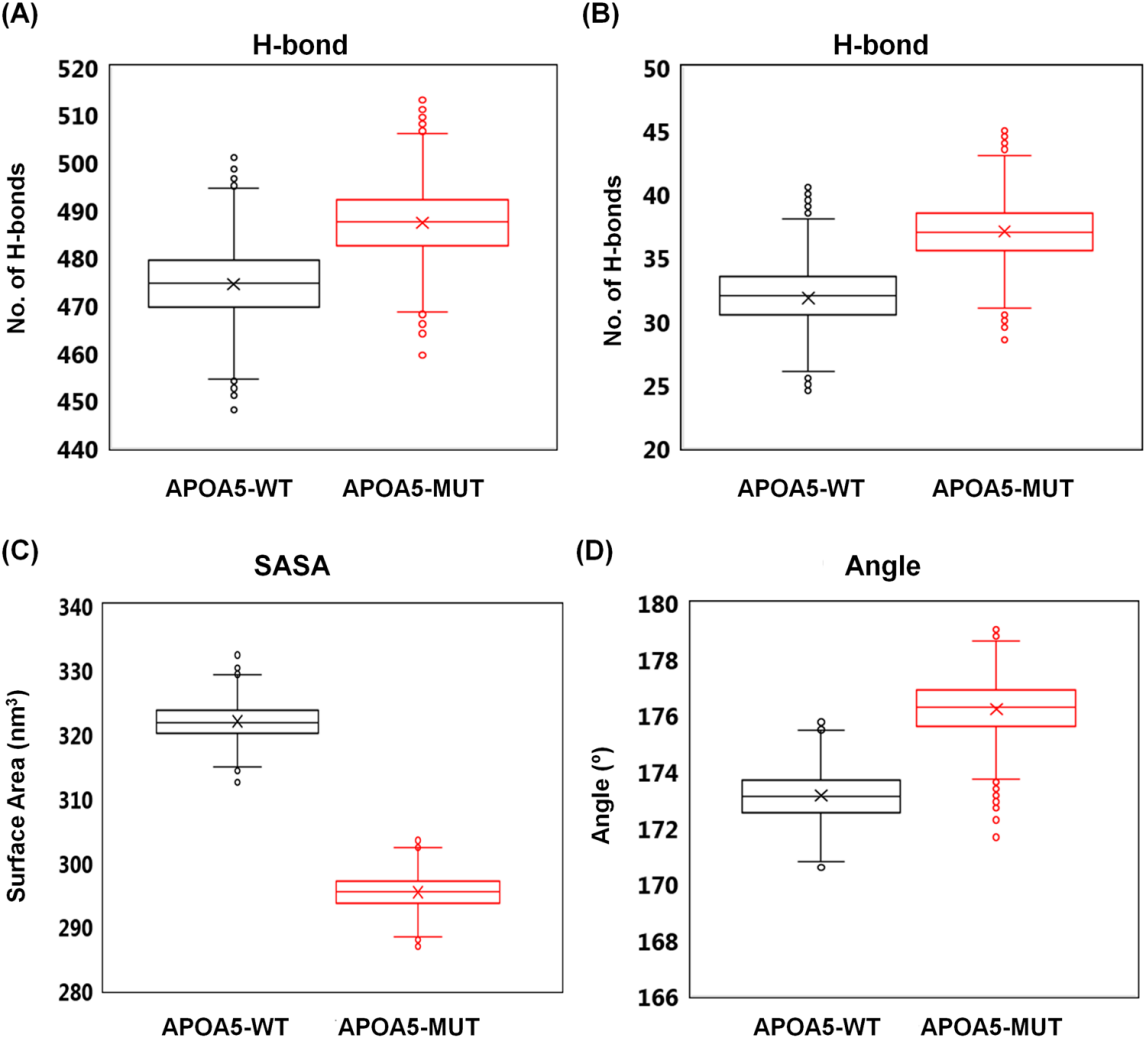
Structural properties of APOA5-WT and APOA5-MUT. The number of hydrogen bonds (H-bonds) within the complex (**A**) and between individual chains (**B**). The SASA with a probe size of 0.14 nm (**C**). The angle along the helical length of APOA5 was calculated to highlight the bendability of APOA5 (**D**). Box plots show the first and third quartile with the median line and mean-marker inside; minimum and maximum values are indicated with lines; outliers are indicated by round markers at the top and bottom of the box. The time-evolved quantities of these analyses are given in Fig. S5.

The numbers of hydrogen bonds (H-bonds) within and between the monomer were significantly different between the WT and mutant protein. APOA5-WT contained lower numbers of inter- and intra-protein H-bonds as compared with APOA5-MUT. These data were suggestive of structural differences between the APOA5-WT and mutant APOA5 protein. The detailed analysis of H-bonds was performed on the average structure of the last nanosecond of each trajectory, and it was found that these H-bonds are scattered along the helical length of the APOA5 protein (Table S3). Mover, solvent-accessible surface area (SASA) also turned out to be larger in APOA5-WT, suggesting that the WT protein has greater solvent exposure (Figs 4 and S5). APOA5 has a long helical structure that fluctuated during the simulation; therefore, the angle along the helical length was calculated to determine the magnitude of protein bending. APOA5-WT showed slightly more bending than APOA5-MUT did, indicating that the G185C substitution in APOA5 stiffens protein structure (Figs 4 and S5).

### Distance and contact density between APOA5 monomers

**Figure 5:**
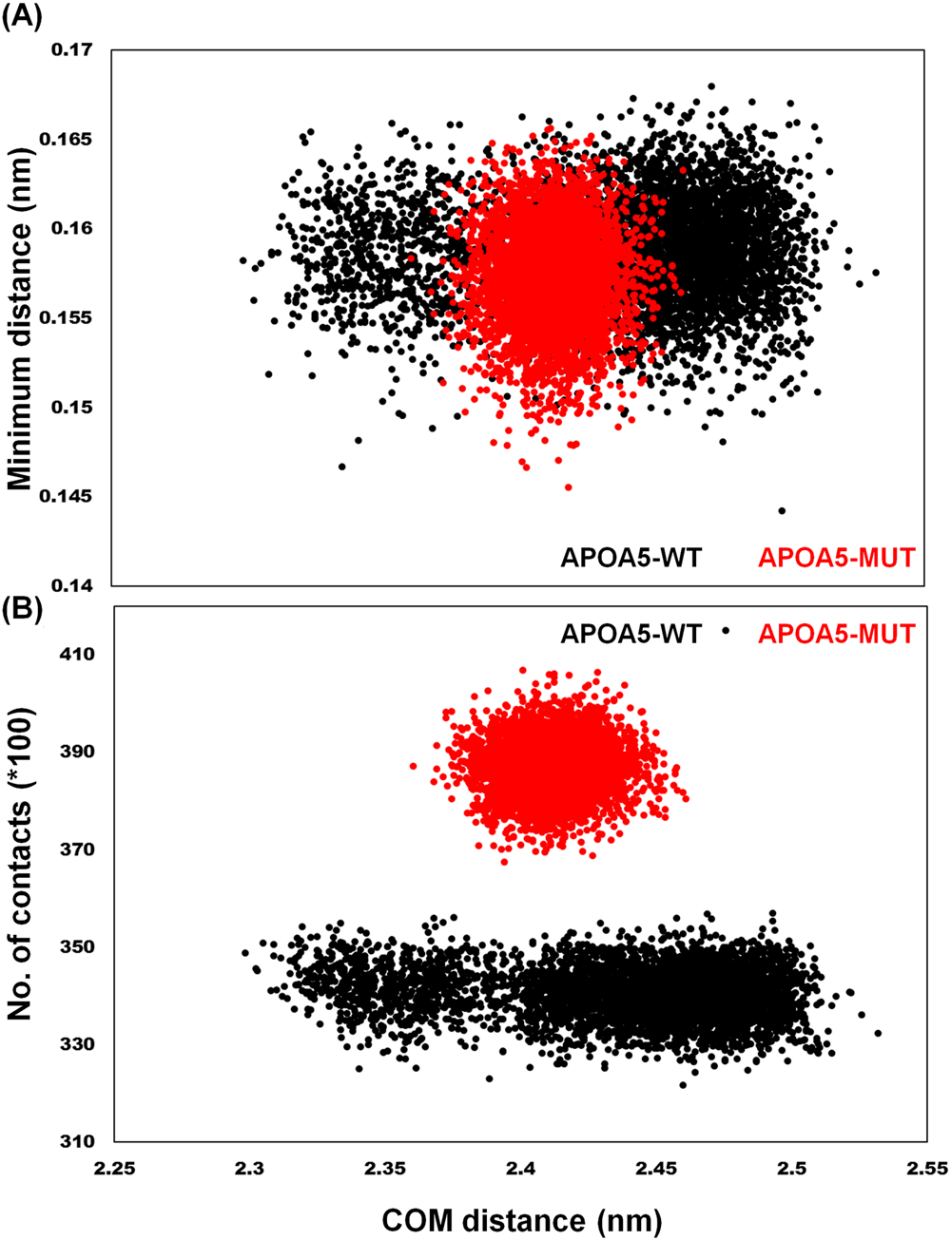
The effect of mutation (G185C) on chain separation. (**A**) The minimum distance between the chains and the average distance between the COMs. (**B**) The number of contacts between the individual chains and COM distance. APOA5-WT showed a COM distance from 2.3 to 2.5 nm with fewer contacts; by contrast, APOA5-MUT showed restricted chain separation, which might have allowed it to establish more contacts.

Given that the mutated protein has a different number of H-bonds and has different root mean square fluctuation (RMSF) values as compared with the WT, we wanted to determine the distance between individual monomers and their relative contacts. The minimum difference between the monomers and the average distance between the centres of mass (COMs) of these monomers were calculated. The results showed that APOA5-WT has a longer average distance between the COMs of its monomers as compared with APOA5-MUT (Fig. 5A). APOA5-MUT was restricted to a shorter range. Similarly, the average number of contacts was lower in APOA5-WT than in the mutant (Fig. 5B).

### Different correlated movements between APOA5-WT and APOA5-MUT

**Figure 6:**
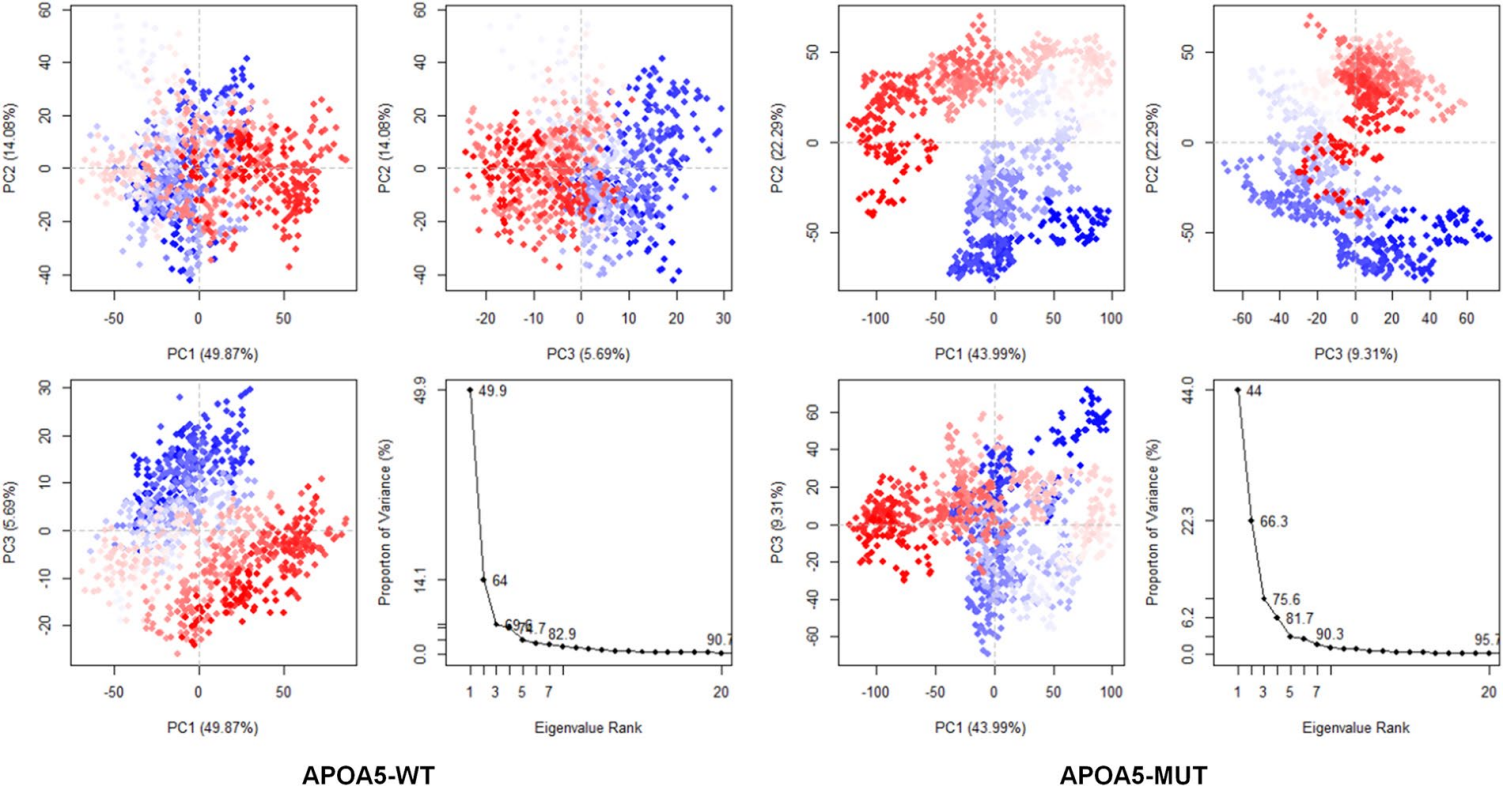
Conformational sampling by PCA. PCA was performed on Cα atoms for 500 snapshots from the last 10 ns using Bio3D library in R v3.2.5. The colours (from blue to white to red) indicate the time of sampling. Continuous coloured dots indicate periodic jumps among these clusters, while a gap between the dots denotes an energy barrier.

Because of the single amino acid change, many striking changes in the properties of the APOA5 complex were observed. Therefore, further analyses were performed to gain an in-depth understanding of structural alterations. To this end, we performed principal component analysis (PCA) on APOA5; the results showed uniform clustering of protein coordinates when PC1/PC2 and PC2/PC3 were compared. PC1/PC3 indicated some different metastable states that had low energy barriers. On the other hand, in the APOA5-MUT complex, the PC1/PC3 comparison did not reveal any significant differences. Furthermore, both PC1/PC2 and PC2/PC3 assumed two metastable states with a higher energy barrier as compared with APOA5-WT (Fig. 6). Based on these results, it is evident that APOA5-WT is more flexible than APOA5-MUT and has greater conformational space.

**Figure 7:**
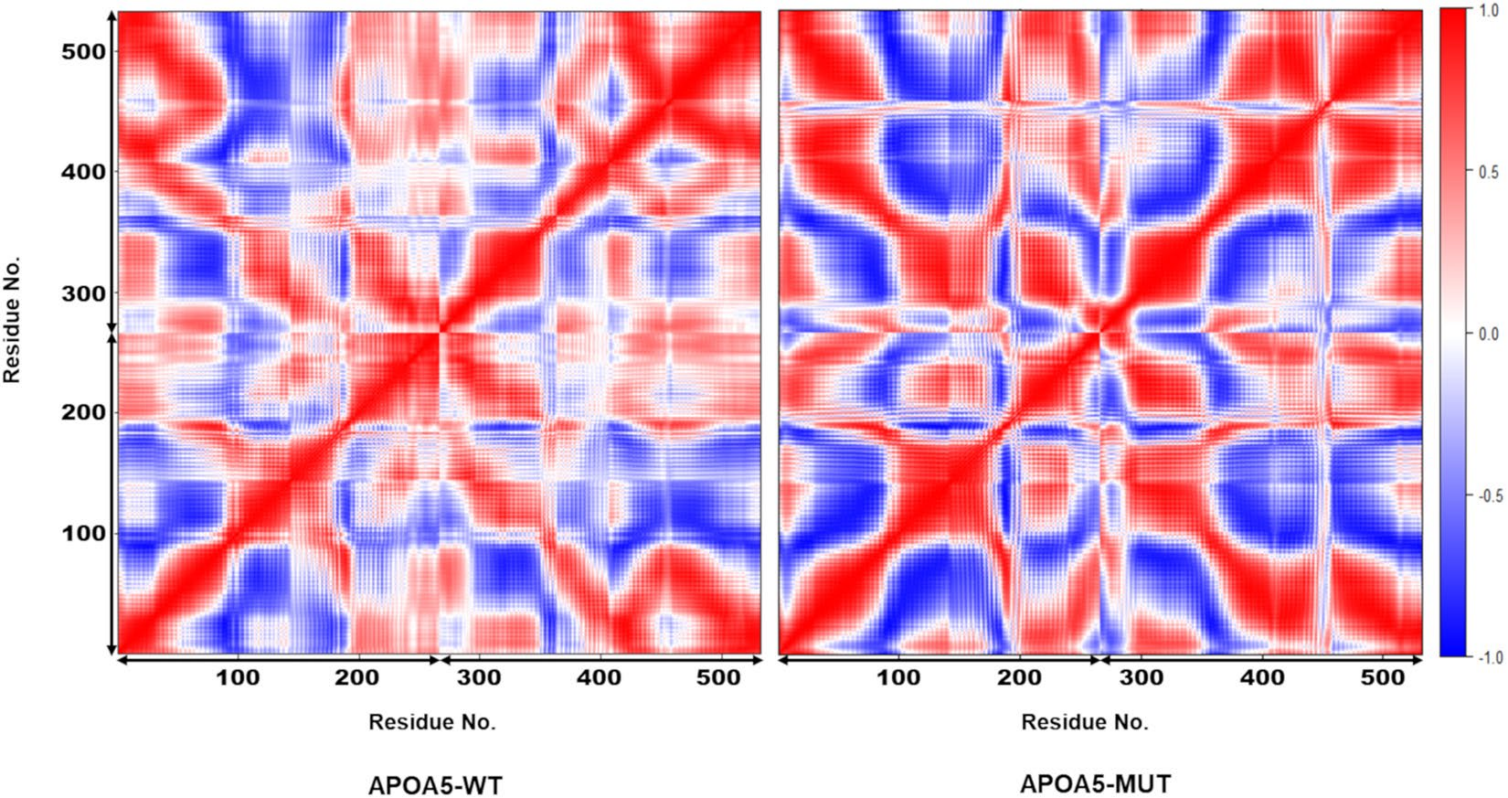
The dynamic cross-correlation matrix. Positive (red) and negative (blue) correlations are defined based on the relative movements of the residues; positive means movement in the same direction, and negative represents the movement in the opposite direction; uncorrelated movement (indicated by white colour) is defined as residue movement that does not influence the movement of others. The diagonal line indicates self-correlation, which is 1.0. This matrix is symmetric; therefore, the lower and upper halves are identical.

When cross-correlation among amino acid residues was plotted, no substantial differences between the two systems were detected; however, several local correlative movements were observed. In general, APOA5-WT showed more non-correlative behaviour than APOA5-MUT, whereas APOA5-MUT manifested strong positive or negative correlative movements (Fig. 7). In addition, this result implied that the mutant complex made restricted and stiffer movements.

## Discussion

In this study, we identified 13 SNPs in a gene cluster, which are independently associated with plasma TG levels. Next, we performed computational analysis of rs2075191, the only SNP that is in a protein-coding region. Our results are in agreement with other reports, about Hong Kong and Guangzhou Chinese subjects. These reports suggested that SNP rs651821 in the promoter region of the *APOA5* gene is significantly associated with TG levels^21^. Our current data also provide strong evidence of associations between the four SNPs, including rs651821, and plasma TG levels among 12,523 individuals in Korea. Furthermore, these results indicated that plasma TG levels may be affected by cumulative effects of multiple variants. Therefore, this highly associative region should be further examined for clinical applications to more precisely define functional variants.

Various studies have revealed that the *BUD13*-*ZNF259*-*APOA5*-*APOA1*-*SIK3* gene cluster is associated with increased plasma TG levels^13–16,22–24^. Results from our study also point to a significant statistical association between this locus and increased plasma TG levels. When these variants were analysed together in our haplotype analysis, three haplotypes (CCACGA, TCACGA, and CCCCGA) showed a strong association with hypertriglyceridaemia. Haplotypes that are associated with higher plasma TG levels involve the risk allele of rs651821 (C); the haplotype that posed the highest risk of elevated plasma TG levels included two risk alleles, rs651821 (C) and rs2075291 (A). As described in Fig. 2, the associations of increased plasma TG levels with rs651821 and rs2075291 were significant and independent of each other. This result suggested that major effects on plasma TG levels may be exerted by the two SNPs: rs651821 and rs2075291.

The aim of our study was to determine the number of genes and functional variants that are involved in TG regulation. Accordingly, we found both harmful and protective variants in the gene cluster that regulates plasma TG levels. The *BUD13*-*ZNF259*-*APOA5*-*APOA1*-*SIK3* gene cluster located in the 11q23.3 chromosomal region encodes a lipoprotein gene: *APOA5*. Therefore, it is rational to assume that *APOA5* is the likely candidate gene that gives rise to these functional variants. Various studies on people of different ancestries have confirmed that *APOA5* (rs2075291 and rs651821) variants are associated with changes in plasma TG levels^11,16,25,26^. The nonsynonymous variant in the *APOA5* coding region (rs2075291, G185C) is mostly found in the Asian population. This specific phenomenon is caused by differences in MAFs between ethnic populations (1% in Caucasians and 7% in Koreans)^27^. Nonetheless, the precise functions of *BUD13*, *ZNF259*, and *SIK3* in TG regulation are unclear. BUD13 is one of the subunits of the splicing factor that participates in nuclear pre-mRNA retention^28^, and ZNF259 is a regulatory protein that is associated with signal transduction and cell proliferation^29^. SIK3 mediates inhibitory effects of cAMP^30^. Nevertheless, *BUD13*, *ZNF259*, and *SIK3* have been reported to be associated with TG levels in many GWASs in both European and Asian populations^13,16,31^. Furthermore, Aung *et al*. have reported that *ZNF259* and *BUD13* are associated with TG levels in two different Chinese populations and showed ethnic or sex specificity^32,33^. The 13 SNPs of this gene cluster are located in noncoding, exon, and intergenic regions. Transcriptional binding sites of nearby genes contribute to transcriptional mechanisms influenced by these SNPs without being directly associated with protein regulation. By contrast, rs2075291 in APOA5 can directly influence the function and structure of APOA5 by offering an opportunity for an additional disulphide bond.

The addition of an extra cysteine in APOA5 can lead to formation of other disulphide bonds with plasma proteins, especially with fibronectin and kininogen-1^19^. This additional bond formation can limit its proper functions. A GWAS on the Taiwanese population uncovered a significant association between this polymorphism and hypertriglyceridaemia (P < 0.001). The increased risk of hypertriglyceridaemia correlated in our study with CC homozygosity; the mean value of TG plasma concentration was low in G185 homozygotes (1.06 mmol/L), moderate in G185C heterozygotes (1.22 mmol/L), and high in C185 homozygotes (21.0 mmol/L). There is a >10-fold higher risk of hypertriglyceridaemia in CC homozygotes^34,35^. Therefore, CC homozygosity significantly correlates with hypertriglyceridaemia. It has also been observed that APOA5-MUT forms multimers *in vitro*; however, it remains monomeric *in vivo*. The nature of this mutation could lead to drastic changes in APOA5 function and conformation due to the position of the mutated glycine in the protein. Of note, there is no difference in the binding of APOA5-MUT to its receptor, LR8, and to low-density lipoprotein receptor-related protein 1 (LRP1)^36^, which suggests that the mutation may affect LPL activation rather than binding. Similarly, MD simulation showed that the mutated protein has stable structure, with differential H-bonding networks, SASA, and contact density that could affect the overall function of the complex rather binding (Fig. 4). Furthermore, the intact 3D structure was also found to be comparable to previous observations^37,38^ (Fig. S4).

The G185C mutation may lead to structural stiffness, as revealed by RMSF (Fig. 3), by lesser bending (Fig. 4D), and the average distance between the monomers and contact density (Fig. 5). These types of structural rigidity can significantly alter the resulting APOA5–receptor complex and reduce activation^39,40^. Similarly, PCA uncovered the existence of energy barriers for various conformations in APOA5-MUT (Fig. 6); these barriers could result in differential correlated motion (Fig. 7). In our model, both systems (APOA5-WT and APOA5-MUT) behaved similarly, as corroborated by the observation that mutated APOA5 binds to its receptor but is unable to initiate the signalling cascade^19^. This binding means that the structure of APOA5 was intact after the mutation; there are subtle conformational changes that resulted in different bonding patterns. Similarly, there might be a few lost hydrogen bonds; this change prevented its proper binding and subsequent receptor activity initiation. In this scenario, our MD simulation results nicely corroborate the experimental results, and further highlight the allosteric nature of protein functions^40^.

By association analysis and molecular modelling, we identified several SNPs in the *BUD13*-*ZNF259*-*APOA5*-*APOA1*-*SIK3* gene cluster that are associated with plasma TG levels in two Korean cohorts. We expect that this finding will help to develop a diagnostic tool for hypertriglyceridaemia, form the basis of functional analysis, and highlight the significance of the *BUD13*-*ZNF259*-*APOA5*-*APOA1*-*SIK3* gene cluster in hypertriglyceridaemia aetiology. In addition, our results suggest that mutated APOA5 might be a potential therapeutic target for TG regulation.

## Methods

### Data sources

We used two independent cohorts in Korea. A total of 3,695 individuals from the Health Examinee (HEXA) study served as the discovery dataset. The Korea Association Resource study (KARE) includes 8,842 individuals from the replication study. The large-scale HEXA study included middle-aged and elderly individuals between 40 and 70 years of age; data were collected in 2004 from participating hospitals, and the study was led by the Korean National Institute of Health. Genome-wide markers were validated in 3,695 participants; six participants whose plasma TG levels were not measured were excluded from analysis. Prospective cohort study KARE was initiated as part of the Korean genome epidemiology study in rural Ansung and urban Ansan in 2001. This study included 10,038 healthy participants between 40 and 69 years of age. Both geographical regions are located in the Gyeonggi Province adjacent to Seoul. Information on the health status and health-related behaviours of the participants was collected through standardised questionnaire surveys. Blood samples were drawn from the antecubital vein following an 8 h fast. Plasma TG levels were measured by an enzymatic method, which was standardised by a centralised laboratory. Genome-wide data were validated in 8,840 participants. Six participants were excluded from the study at the time of analysis because two of them did not undergo measurement of plasma TG levels, and the other four participants did not measure body weight, whereas data obtained from rest of the 8,834 participants were used for the analysis. Detailed explanations of KARE have been reported in other studies^41,42^. The information about KoGES (Korean Genome Epidemiology Study) can be viewed at [http://www.nih.go.kr/NIH/eng/main.jsp > Research infrastructure > KoGES]. The detailed cohort profile has been reported in another study^43^.

All individuals voluntarily participated in this study, and all the participants provided written informed consent. The study was conducted in accordance with the guidelines authorised by the Ethics Committee of KoGES at the Korean National Institute of Health. The protocol used for this project was approved by the Institutional Review Board (IRB) of Ajou University (201612-HB-002).

### Measurement of anthropometric and biochemical parameters

The BMI was calculated (weight divided by height squared, kg/m^2^), followed by the measurement of body weight and height via standard methods in light clothing. The circulation levels of TG, TCHL, and glucose were measured after a 12 h fast on a Hitachi 747 chemistry analyser (Hitachi Ltd, Tokyo, Japan).

### Quality control (QC) and genotyping

Both studies used genome-wide condensed SNP marker analysis; HEXA used the Affymetrix Genome-Wide Human SNP Array GeneChip 6.0, and KARE utilised the Affymetrix Genome-Wide Human SNP Array GeneChip 5.0. For HEXA, markers with significant deviation from the Hardy–Weinberg equilibrium (P < 10^−6^), missing genotype rate over 5%, and MAF <0.01 were excluded; the remaining 627,659 markers were examined. Based on the same criteria as described above, 352,228 markers were used for analysis of KARE data. Imputation in HEXA was performed by means of IMPUTE2 (http://mathgen.stats.ox.ac.uk/impute/impute.html)^44^ and SHAPEIT software (https://mathgen.stats.ox.ac.uk/genetics_software/shapeit/shapeit.html)^45^. Using 1000 Genomes Project’s haplotype phase I in NCBI build 37 (hg19), only markers with imputation certainty score >0.9 were chosen for subsequent analysis. In KARE, the reference panel originated from the 1000 Genomes Project’s haplotype phase I in NCBI build 37 (hg19). As a result of imputation, 4,166,520 and 3,512,376 markers were selected from KARE and HEXA, respectively.

### Statistical analysis

The effects of genotypes on plasma TG levels were evaluated via mixed linear models with adjustment for age, sex, and the BMI. For our association study, statistical analysis was performed in the PLINK software^46^. TG–SNP associations as well as their concomitant betas and conditional analysis were assessed using the GCTA software^47,48^. In the conditional analysis, a stepwise model selection procedure was performed to select independently associated SNPs. Haplotype analysis was conducted by means of the haplo.stats package in the R software, version 3.4.1 (http://www.r-project.org/)^49^. Haplotypes with frequencies <0.01 were excluded from the analysis. LD matrix analysis for CHB + JPT and CEU was carried out in LDlink (https://analysistools.nci.nih.gov/LDlink/)^50^. Box plots were constructed and *P* values were calculated using the SPSS 24 software (SPSS Inc., Chicago, Illinois).

### Molecular modelling and MD simulation

Because there was no crystal structure available, a 3D model of APOA5 was created through different protocols including homology modelling and threading. For the homology model, the protein sequence of APOA5 was searched against the Protein Data Bank (PDB) database by means of the protein-BLAST tool with default parameters. APOA-IV (PDB code 3S84^51^) was found to have approximately 30% identity and 51% sequence similarity (without 84 N-terminal and 42 C-terminal residues) and belongs to the same family as APOA5. Furthermore, the sequence of APOA-5 was aligned against the available template of APOA-IV (3S83) in Clustal Omega^52^, and a homology model was built for amino acid residues 78–343; energy was minimised using the homology modelling suite in Molecular Operating Environment (MOE) (Chemical Computing Group, Montreal) and MODELLER^53^ v9.17. To complete the model, we tried to build N- and C-terminal parts by ab initio methods, but it was challenging to adjust the relative orientation of those parts, and MODELLER lacks this capability. Because our desired polymorphic region falls into the modelled region, we focused on this region. Furthermore, model quality was evaluated on the ProSA web server^54^ and the Ramachandran plot (>97% residues were in the allowed region, and only 11 residues were outliers). We found that homology models from MOE yielded better-quality models that kept their 3D structure after energy minimisation and were better suited for dimeric forms. For threading-based modelling, I-TASSER^55^ was employed for the whole protein sequence. Nonetheless, the resultant models were either non-biological or of poor quality. Therefore, we proceeded with the homology models.

Docking simulations were performed by means of PatchDock^56^, Rosie Dock^57^, and MOE docking suites. We used a reference dimer structure as the literature suggested because this protein forms a multimeric biological entity^36,58^. The resulting docking solutions were cross-validated via the following criteria: (1) the dimer should be anti-parallel so that the monomers fit snugly to each other, (2) and the position of C185 with respect to the dimer. Among the top 10 poses, the number of anti-parallel poses was greater (>60%) in each docking experiment. The anti-parallel poses were further evaluated for possible disulphide bond formation. We noted that C185 was consistently on the outer edge of the dimer and this arrangement allows it to form potential disulphide bonds with other proteins. To generate the final dimer model, a combination of algorithms (such as MOE, PatchDock, and Rosie Dock), and a literature search^36,58,59^ were utilised. After careful evaluation of docking poses, a final model was selected that showed lesser Cα-RMSD (root-mean square deviation) relative to that of 3S84 (3.27 Å for Cα), and we termed this model as APOA5-WT. APOA5-MUT was created by substituting G185 with cysteine, and both complexes were then studied by MD simulations.

### MD simulations

These simulations were performed via the CHARMm22 (with CMAP correction)^60^ forcefield definition in GROMACS^61^ v2018.1. A dodecahedron box was created around the proteins, filled with the TIP3P water model^62^, and neutralised with the counter-ions Na^+^ and Cl^−^; and adjusted the ionic concentration of 0.1 M. The systems were energy-minimised through the steepest descent, and if a system did not converge to <10 kJ · mol^−1^ · nm^−1^, a conjugate gradient with a step size of 0.01 nm was applied. The systems were then equilibrated in a two-step manner. During the first 100 ps, temperature was equilibrated at 300 K by the V-Rescale algorithm^63^. The second step involved pressure equilibration at 1 atm via the Parrinello–Rahman algorithm^64^. At both steps, all atoms were restrained to avoid any physical distortion.

During production simulations, for integrating Newton’s equation, the leap-frog integrator was used; only H-bonds were restrained by means of LINCS^65^. A 1.2 nm cut-off for short-range electrostatics and Van der Waals forces were applied, with force switches at 1.0 nm. The particle mesh Ewald method was employed for calculating long-range electrostatics^66^. For both systems, two independent simulations were carried out at different starting velocities, and the results were the average of these simulation runs. The coordinates and energy terms after every 2 ps were saved for analysis, which was mostly carried out with GROMACS built-in tools. Nevertheless, PCA plots and dynamic cross-correlation matrix were created via the Bio3D library^67^ in the R software. Figures were created in PyMol viewer (The PyMOL Molecular Graphics System, Version 1.7.4 Schrödinger, LLC), and charts were created in Xmgrace and MS Excel (2016).

## Supplementary information

Supporting Information
