# Supplementary material for: Association of BUD13-ZNF259-APOA5-APOA1-SIK3 cluster polymorphism in 11q23.3 and structure of APOA5 with increased plasma triglyceride levels in a Korean population
Source: Sci Rep. 2019 Jun 5;9:8296. doi: 10.1038/s41598-019-44699-x (PMC6549162; doi:10.1038/s41598-019-44699-x)
Supplement: Supplementary file 1 — Supporting Information [file 41598_2019_44699_MOESM1_ESM.pdf]

## < Supporting Information >

**Association of *BUD13-ZNF259-APOA5-APOA1-SIK3* cluster polymorphism in 11q23.3 and structure of APOA5 with increased plasma triglyceride levels in a Korean population**

Han-Kyul Kim[1], Muhammad Ayaz Anwar[1] & Sangdun Choi[1*]

[1]Department of Molecular Science and Technology, Ajou University, Suwon, 16499, Korea

*Corresponding author:

Sangdun Choi

Department of Molecular Science and Technology, Ajou University, Suwon, 16499, Korea

Phone: +82-31-219-2600

Fax: +82-31-219-1615

E-mail: sangdunchoi@ajou.ac.kr

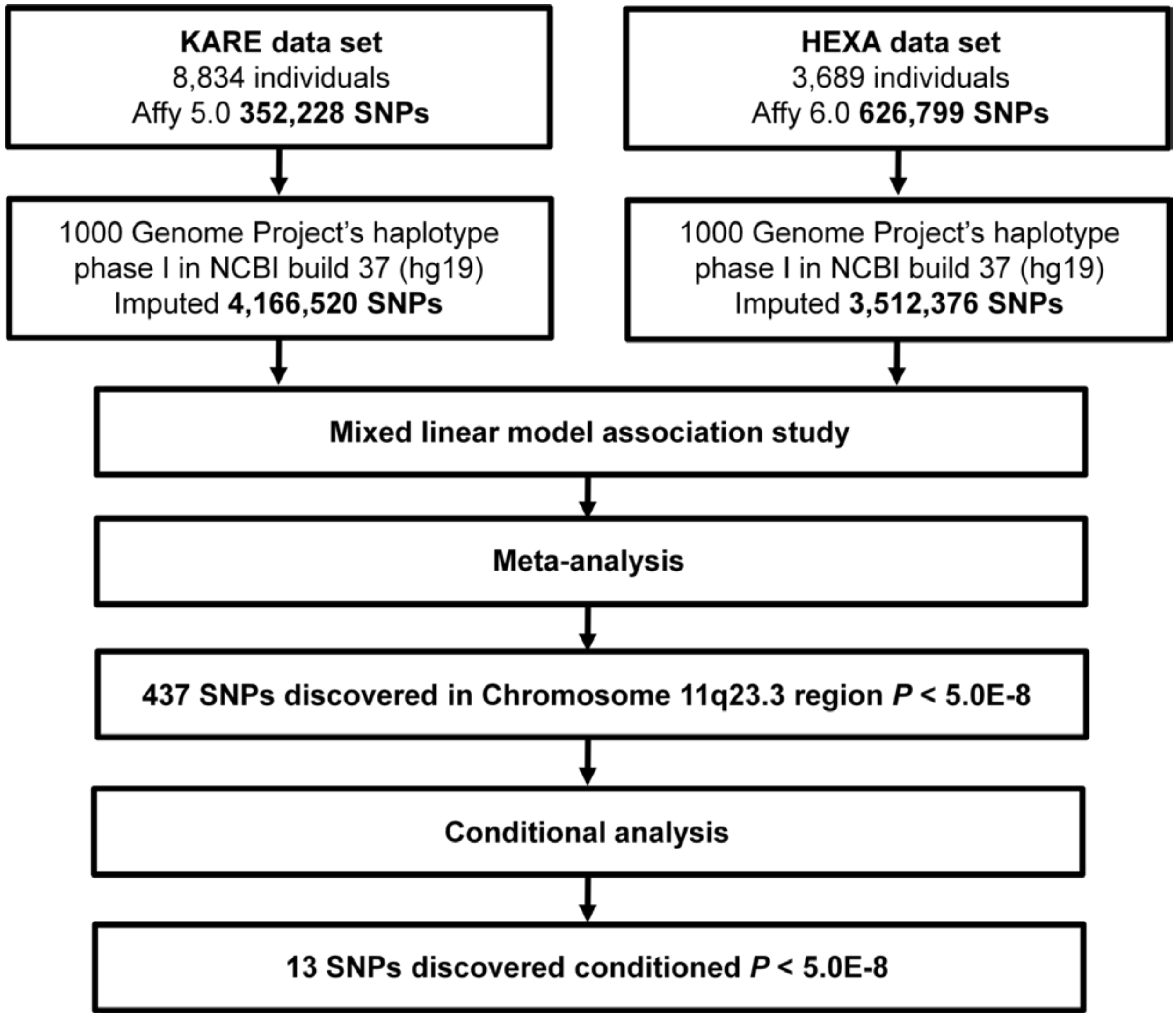


**S1 Fig. Overall study design.**

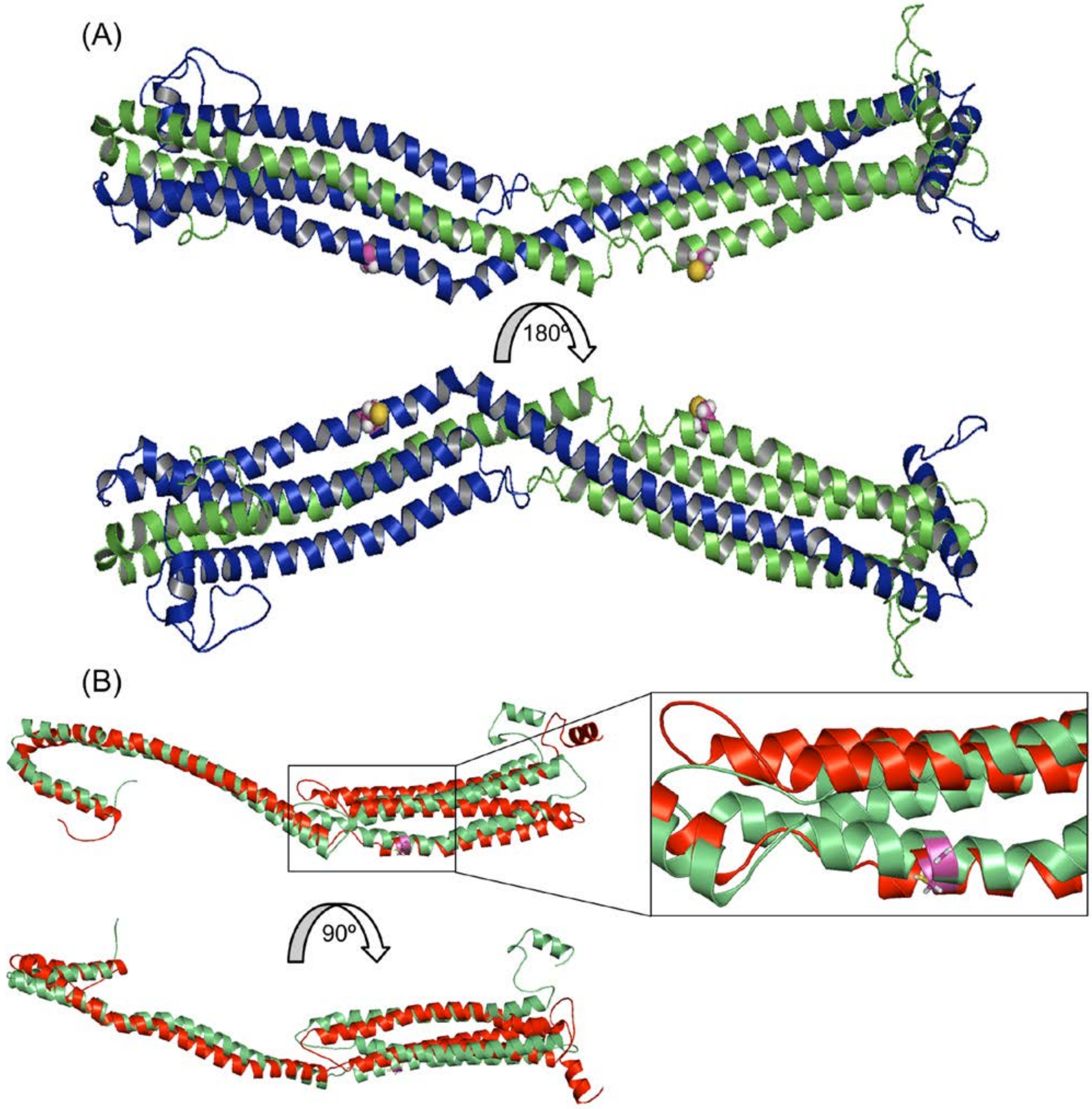


**S2 Fig. Structural organization of APOA5.** (A) Homology model and dimer of APOA5; chains are differentially colored (green and blue). The mutated form has been created by mutating the side chain of glycine into cysteine at position 185, followed by energy minimization. Since the overall structure and organization did not change between APOA5-WT and APOA5-MUT, only APOA5-MUT was shown with C185 in the sphere (Cβ in pink, S in yellow, Hs in white). (B) The comparison of

APOA5-WT (green) and APOA5-MUT (red) after 100 ns of molecular dynamics simulations. The models are averages of the last 2 ns of each trajectory and have been aligned to highlight the differences. Only single chains are shown for clarity. Glycine and cysteine are shown in stick organization, with the same color assignment as A. The inset shows a zoomed-in image of the mutated site; displacement of helices, relative angle differences, and the extended loop in the APOA5-MUT complex can be observed.

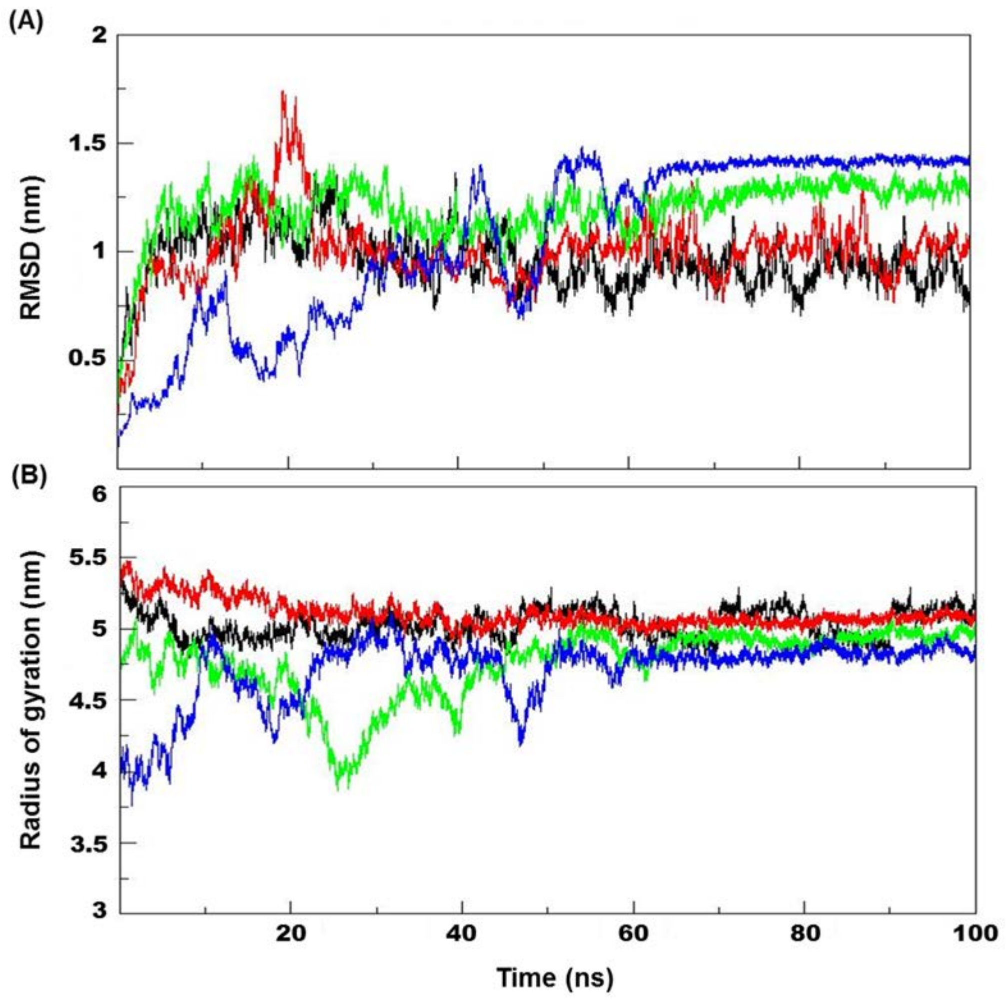


**S3 Fig. Structural properties of APOA5.** (A) Root mean square deviation of APOA5-WT and APOA5-MUT over the length of individual trajectories. The first and second trajectories APOA5-WT are represented by red and black lines, respectively. The first and second APOA5-MUT trajectories are represented by blue and green, respectively. (B) The radius of gyration (Rg) is plotted on the y-axis, and time is plotted along x-axis. The color scheme is same as that for RMSD.

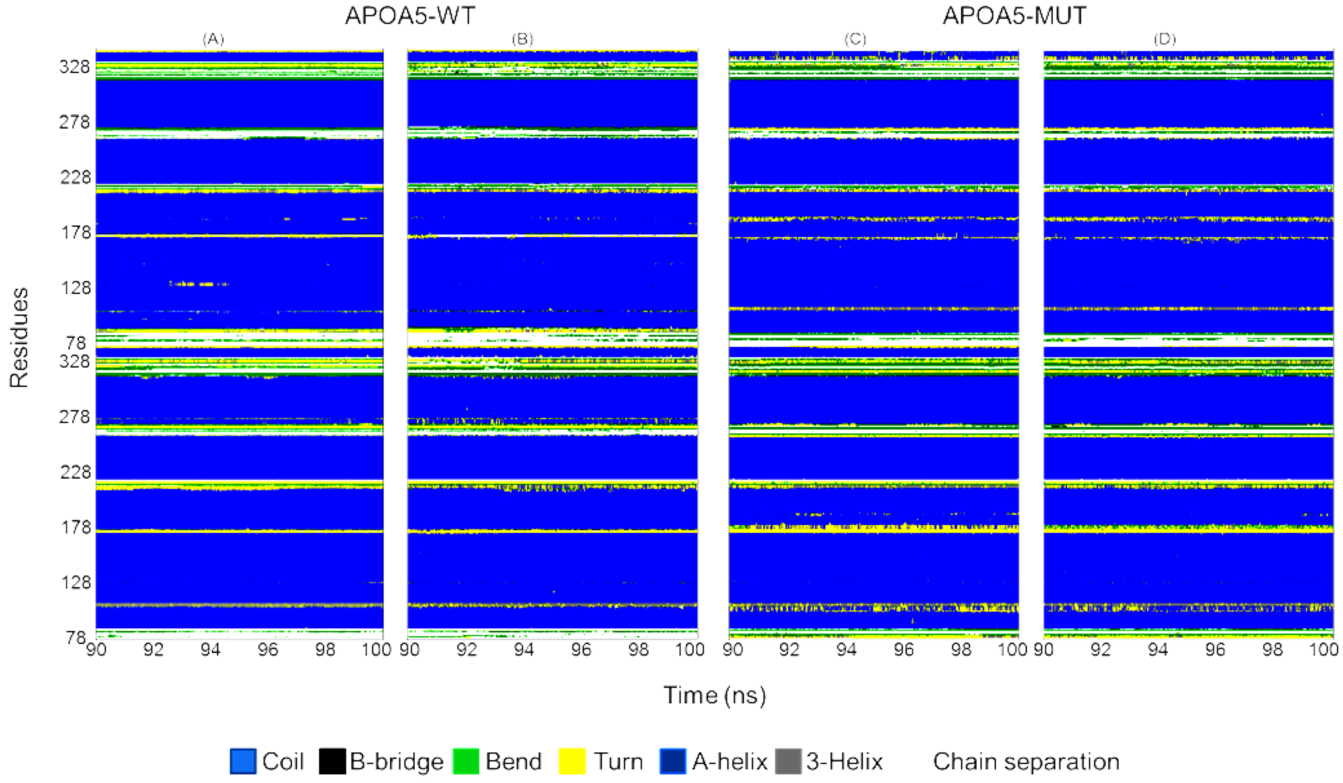


**S4 Fig. Secondary structure evolution of APOA5.** Secondary structures were calculated as per definition of secondary structure of proteins (DSSP) classification. The DSSP for APOA5-WT (A and B) and APOA5-MUT (C and D) in the last 10 ns of each trajectory are given. The legend indicates individual secondary structures.

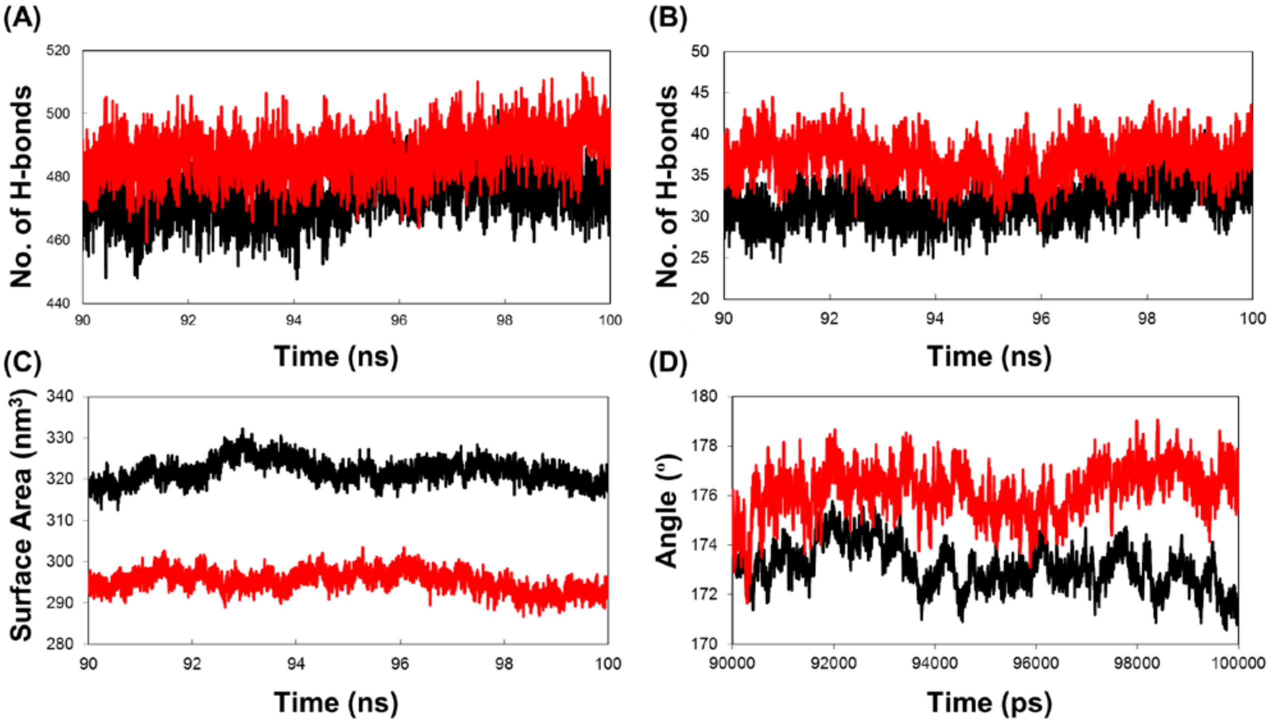


**S5 Fig. Structural properties of APOA5 complexes.** The time evolved number of hydrogen bonds (H-bonds) (A) within proteins, and (B) between the chains of complex is shown. (C) solvent accessible surface area (C); (D) the angle along the length of protein (D). Data is given as the average of the last 10 ns from two independent simulations. APOA5-WT and APOA5-MUT are indicated with black and red lines, respectively.

**S1 Table.** Detailed linkage disequilibrium matrix among the 33 SNPs in the BUD13-ZNF259-APOA5 gene cluster.

| | rs184616707 | rs118175510 | rs60954647 | rs79408961 | rs180373 | rs11604424 | rs2075291 | rs651821 | rs7123666 | rs525028 | rs645258 | rs10160754 | rs142395187 | |
|---|---|---|---|---|---|---|---|---|---|---|---|---|---|---|
| rs184616707 | 1.000 | 0.001 | 0.009 | 0.000 | 0.001 | 0.002 | 0.064 | 0.017 | 0.000 | 0.001 | 0.006 | 0.002 | 0.001 | **Korean** |
| rs118175510 | 0.001 | 1.000 | 0.102 | 0.007 | 0.014 | 0.106 | 0.003 | 0.260 | 0.158 | 0.029 | 0.025 | 0.013 | 0.015 | |
| rs60954647 | 0.009 | 0.102 | 1.000 | 0.074 | 0.083 | 0.025 | 0.066 | 0.150 | 0.043 | 0.061 | 0.003 | 0.015 | 0.001 | |
| rs79408961 | 0.000 | 0.007 | 0.074 | 1.000 | 0.007 | 0.065 | 0.273 | 0.026 | 0.012 | 0.006 | 0.023 | 0.004 | 0.008 | |
| rs180373 | 0.001 | 0.014 | 0.083 | 0.007 | 1.000 | 0.114 | 0.006 | 0.042 | 0.018 | 0.004 | 0.011 | 0.025 | 0.027 | |
| rs11604424 | 0.002 | 0.106 | 0.025 | 0.065 | 0.114 | 1.000 | 0.076 | 0.109 | 0.165 | 0.138 | 0.070 | 0.009 | 0.077 | |
| rs2075291 | 0.064 | 0.003 | 0.066 | 0.273 | 0.006 | 0.076 | 1.000 | 0.189 | 0.012 | 0.007 | 0.000 | 0.014 | 0.005 | |
| rs651821 | 0.017 | 0.260 | 0.150 | 0.026 | 0.042 | 0.109 | 0.189 | 1.000 | 0.388 | 0.068 | 0.060 | 0.045 | 0.032 | |
| rs7123666 | 0.000 | 0.158 | 0.043 | 0.012 | 0.018 | 0.165 | 0.012 | 0.388 | 1.000 | 0.034 | 0.182 | 0.017 | 0.021 | |
| rs525028 | 0.001 | 0.029 | 0.061 | 0.006 | 0.004 | 0.138 | 0.007 | 0.068 | 0.034 | 1.000 | 0.108 | 0.042 | 0.175 | |
| rs645258 | 0.006 | 0.025 | 0.003 | 0.023 | 0.011 | 0.070 | 0.000 | 0.060 | 0.182 | 0.108 | 1.000 | 0.076 | 0.051 | |
| rs10160754 | 0.002 | 0.013 | 0.015 | 0.004 | 0.025 | 0.009 | 0.014 | 0.045 | 0.017 | 0.042 | 0.076 | 1.000 | 0.455 | |
| rs142395187 | 0.001 | 0.015 | 0.001 | 0.008 | 0.027 | 0.077 | 0.005 | 0.032 | 0.021 | 0.175 | 0.051 | 0.455 | 1.000 | |

**CHB+JPT**

| | | | | | | | | | | | | | |
|---|---|---|---|---|---|---|---|---|---|---|---|---|---|
| rs184616707 | 1.000 | 0.001 | 0.004 | 0.000 | 0.001 | 0.004 | 0.150 | 0.011 | 0.001 | 0.002 | 0.009 | 0.001 | 0.001 |
| rs118175510 | 0.001 | 1.000 | 0.092 | 0.009 | 0.013 | 0.085 | 0.004 | 0.161 | 0.039 | 0.020 | 0.008 | 0.004 | 0.003 |
| rs60954647 | 0.004 | 0.092 | 1.000 | 0.052 | 0.059 | 0.001 | 0.026 | 0.152 | 0.092 | 0.077 | 0.018 | 0.010 | 0.000 |
| rs79408961 | 0.000 | 0.009 | 0.052 | 1.000 | 0.009 | 0.058 | 0.193 | 0.001 | 0.009 | 0.015 | 0.015 | 0.018 | 0.011 |
| rs180373 | 0.001 | 0.013 | 0.059 | 0.009 | 1.000 | 0.103 | 0.004 | 0.051 | 0.019 | 0.001 | 0.014 | 0.019 | 0.026 |
| rs11604424 | 0.004 | 0.085 | 0.001 | 0.058 | 0.103 | 1.000 | 0.029 | 0.300 | 0.230 | 0.202 | 0.103 | 0.021 | 0.057 |
| rs2075291 | 0.150 | 0.004 | 0.026 | 0.193 | 0.004 | 0.029 | 1.000 | 0.073 | 0.009 | 0.011 | 0.000 | 0.005 | 0.003 |
| rs651821 | 0.011 | 0.161 | 0.152 | 0.001 | 0.051 | 0.300 | 0.073 | 1.000 | 0.514 | 0.132 | 0.179 | 0.057 | 0.032 |
| rs7123666 | 0.001 | 0.039 | 0.092 | 0.009 | 0.019 | 0.230 | 0.009 | 0.514 | 1.000 | 0.076 | 0.341 | 0.037 | 0.022 |
| rs525028 | 0.002 | 0.020 | 0.077 | 0.015 | 0.001 | 0.202 | 0.011 | 0.132 | 0.076 | 1.000 | 0.137 | 0.086 | 0.146 |
| rs645258 | 0.009 | 0.008 | 0.018 | 0.015 | 0.014 | 0.103 | 0.000 | 0.179 | 0.341 | 0.137 | 1.000 | 0.079 | 0.048 |
| rs10160754 | 0.001 | 0.004 | 0.010 | 0.018 | 0.019 | 0.021 | 0.005 | 0.057 | 0.037 | 0.086 | 0.079 | 1.000 | 0.638 |
| rs142395187 | 0.001 | 0.003 | 0.000 | 0.011 | 0.026 | 0.057 | 0.003 | 0.032 | 0.022 | 0.146 | 0.048 | 0.638 | 1.000 |

**CEU**

| | | | | | | | | | | | | | |
|---|---|---|---|---|---|---|---|---|---|---|---|---|---|
| rs184616707 | NA | NA | NA | NA | NA | NA | NA | NA | NA | NA | NA | NA | NA |
| rs118175510 | NA | NA | NA | NA | NA | NA | NA | NA | NA | NA | NA | NA | NA |
| rs60954647 | NA | NA | 1.000 | NA | 0.241 | 0.126 | NA | 0.051 | 0.068 | 0.068 | 0.039 | 0.004 | 0.004 |
| rs79408961 | NA | NA | NA | NA | NA | NA | NA | NA | NA | NA | NA | NA | NA |
| rs180373 | NA | NA | 0.241 | NA | 1.000 | 0.003 | NA | 0.026 | 0.025 | 0.004 | 0.015 | 0.000 | 0.007 |
| rs11604424 | NA | NA | 0.126 | NA | 0.003 | 1.000 | NA | 0.304 | 0.165 | 0.396 | 0.135 | 0.006 | 0.010 |
| rs2075291 | NA | NA | NA | NA | NA | NA | NA | NA | NA | NA | NA | NA | NA |
| rs651821 | NA | NA | 0.051 | NA | 0.026 | 0.304 | NA | 1.000 | 0.656 | 0.256 | 0.561 | 0.005 | 0.003 |
| rs7123666 | NA | NA | 0.068 | NA | 0.025 | 0.165 | NA | 0.656 | 1.000 | 0.302 | 0.554 | 0.001 | 0.005 |
| rs525028 | NA | NA | 0.068 | NA | 0.004 | 0.396 | NA | 0.256 | 0.302 | 1.000 | 0.263 | 0.003 | 0.012 |
| rs645258 | NA | NA | 0.039 | NA | 0.015 | 0.135 | NA | 0.561 | 0.554 | 0.263 | 1.000 | 0.008 | 0.005 |
| rs10160754 | NA | NA | 0.004 | NA | 0.000 | 0.006 | NA | 0.005 | 0.001 | 0.003 | 0.008 | 1.000 | 0.568 |
| rs142395187 | NA | NA | 0.004 | NA | 0.007 | 0.010 | NA | 0.003 | 0.005 | 0.012 | 0.005 | 0.568 | 1.000 |

**S2 Table.** Top 426 SNPs that satisfied genome-wide significance ($P < 5.0 \times 10^{-8}$) after meta-analysis.

| CHR | Position | SNP ID | Effect allele | Combined P | Beta |
|---|---|---|---|---|---|
| 11 | 116662579 | rs651821 | C | 4.91E-100 | 29.25 |
| 11 | 116663707 | rs662799 | G | 6.57E-100 | 29.47 |
| 11 | 116661392 | rs2075291 | A | 9.60E-67 | 40.53 |
| 11 | 116657667 | rs3741297 | T | 2.82E-66 | 40.36 |
| 11 | 116622299 | rs74368849 | A | 8.56E-66 | 40.58 |
| 11 | 116649806 | rs75198898 | A | 6.50E-65 | 40.07 |
| 11 | 116650638 | rs113932726 | T | 6.94E-65 | 40.05 |
| 11 | 116599535 | rs76240380 | A | 1.53E-44 | 40.35 |
| 11 | 116596894 | rs75662937 | T | 2.03E-44 | 40.40 |
| 11 | 116653296 | rs2075290 | C | 1.53E-35 | 19.05 |
| 11 | 116652423 | rs6589566 | G | 1.88E-35 | 18.96 |
| 11 | 116656788 | rs10750096 | C | 2.00E-35 | 18.98 |
| 11 | 116661826 | rs2072560 | T | 3.27E-35 | 18.98 |
| 11 | 116652491 | rs7483863 | A | 3.65E-35 | 18.89 |
| 11 | 116660686 | rs2266788 | G | 6.58E-35 | 18.84 |
| 11 | 116611827 | rs180349 | A | 7.41E-33 | 18.38 |
| 11 | 116624153 | rs6589564 | C | 1.41E-32 | 18.36 |
| 11 | 116611733 | rs9326246 | C | 1.62E-32 | 18.31 |
| 11 | 116606766 | rs1974718 | G | 4.27E-32 | 18.09 |
| 11 | 116607437 | rs1558861 | C | 4.95E-32 | 18.16 |
| 11 | 116617782 | rs180339 | T | 2.25E-29 | -14.41 |
| 11 | 116628401 | rs2075295 | C | 3.11E-29 | -14.40 |
| 11 | 116626735 | rs180322 | C | 1.10E-25 | -13.65 |
| 11 | 116647120 | rs11216136 | G | 1.11E-25 | -15.98 |
| 11 | 116619605 | rs34637368 | A | 1.14E-25 | -16.61 |
| 11 | 116620256 | rs11216129 | A | 2.24E-25 | -16.34 |
| 11 | 116625875 | rs12421652 | T | 3.55E-25 | -16.29 |
| 11 | 116610294 | rs61905084 | C | 6.08E-25 | -14.71 |
| 11 | 116617240 | rs11216126 | C | 6.37E-25 | -16.14 |
| 11 | 116652301 | rs4417316 | T | 6.51E-25 | -15.59 |
| 11 | 116651463 | rs1942478 | G | 6.54E-25 | -15.61 |
| 11 | 116634557 | rs17119975 | C | 7.02E-25 | -16.07 |
| 11 | 116627055 | rs4938309 | T | 1.05E-24 | -16.11 |
| 11 | 116632798 | rs11602240 | C | 1.06E-24 | -16.11 |
| 11 | 116640094 | rs610675 | A | 1.18E-24 | -15.60 |
| 11 | 116640368 | rs623908 | G | 1.18E-24 | -15.60 |

| | | | | | |
|---|---|---|---|---|---|
| 11 | 116612274 | rs11216125 | A | 1.18E-24 | -16.10 |
| 11 | 116640062 | rs622604 | C | 1.46E-24 | -15.57 |
| 11 | 116646062 | rs11216135 | G | 1.52E-24 | -15.58 |
| 11 | 116646858 | rs11602073 | T | 1.56E-24 | -15.58 |
| 11 | 116639692 | rs1268353 | T | 2.70E-24 | -15.50 |
| 11 | 116603677 | rs1893460 | A | 1.93E-23 | -14.45 |
| 11 | 116604514 | rs4938307 | A | 2.76E-23 | -15.98 |
| 11 | 116598248 | rs10892023 | T | 3.71E-23 | 13.61 |
| 11 | 116603134 | rs2008915 | C | 4.02E-23 | 13.68 |
| 11 | 116601945 | rs3212282 | A | 8.94E-23 | 13.57 |
| 11 | 116654435 | rs603446 | T | 9.31E-22 | -14.51 |
| 11 | 116612659 | rs180346 | C | 1.19E-21 | -14.38 |
| 11 | 116618024 | rs11216127 | G | 2.32E-21 | -14.00 |
| 11 | 116631391 | rs3741301 | C | 6.65E-21 | -14.24 |
| 11 | 116596174 | rs180365 | T | 7.41E-21 | -13.95 |
| 11 | 116610048 | rs180350 | T | 9.34E-21 | -14.14 |
| 11 | 116664776 | rs1787680 | A | 4.47E-19 | -14.03 |
| 11 | 116580869 | rs75476300 | C | 6.89E-19 | 20.62 |
| 11 | 116667083 | rs7123666 | A | 9.29E-19 | 15.99 |
| 11 | 116588593 | rs79408961 | T | 1.01E-17 | 20.23 |
| 11 | 116665079 | rs9804646 | T | 1.63E-17 | -14.69 |
| 11 | 116623659 | rs180327 | C | 1.49E-16 | 10.68 |
| 11 | 116651115 | rs11604424 | C | 7.13E-14 | 9.52 |
| 11 | 116715784 | rs142174850 | A | 6.28E-13 | -16.32 |
| 11 | 116726709 | rs112618266 | T | 6.44E-13 | -16.27 |
| 11 | 116756183 | rs2044426 | A | 6.46E-13 | -15.96 |
| 11 | 116963277 | rs74420345 | A | 6.90E-13 | -14.84 |
| 11 | 116747282 | rs17120119 | G | 7.47E-13 | -15.90 |
| 11 | 116754265 | rs12416987 | C | 8.03E-13 | -15.91 |
| 11 | 116756986 | rs10892040 | A | 8.03E-13 | -15.91 |
| 11 | 116757248 | rs11216174 | G | 8.03E-13 | -15.91 |
| 11 | 116757398 | rs11216175 | T | 8.03E-13 | -15.91 |
| 11 | 116757793 | rs12284346 | C | 8.03E-13 | -15.91 |
| 11 | 116962661 | rs116861530 | A | 8.06E-13 | -14.79 |
| 11 | 116748744 | rs12286581 | C | 8.98E-13 | -15.88 |
| 11 | 116749072 | rs75294699 | A | 8.98E-13 | -15.88 |
| 11 | 116748169 | rs12278117 | A | 9.26E-13 | -15.87 |
| 11 | 116748515 | rs12292614 | G | 9.26E-13 | -15.87 |
| 11 | 116748221 | rs12279433 | T | 9.47E-13 | -15.86 |
| 11 | 116576415 | rs1263056 | A | 1.01E-12 | -9.90 |
| 11 | 116583513 | rs886022 | A | 1.35E-12 | -11.41 |
| 11 | 116735460 | rs17120099 | C | 1.38E-12 | -16.04 |
| 11 | 116657561 | rs3741298 | C | 1.73E-12 | 8.96 |

| | | | | | |
|---|---|---|---|---|---|
| 11 | 116947306 | rs12418036 | A | 1.88E-12 | -14.45 |
| 11 | 116519655 | rs516226 | T | 1.90E-12 | 9.57 |
| 11 | 116584317 | rs1144035 | C | 1.98E-12 | -11.33 |
| 11 | 117003459 | rs17120373 | G | 2.10E-12 | -12.49 |
| 11 | 116663851 | rs17120035 | T | 2.34E-12 | -16.00 |
| 11 | 116663966 | rs7103224 | A | 2.34E-12 | -16.00 |
| 11 | 116972227 | rs148233183 | A | 2.50E-12 | -14.85 |
| 11 | 116936871 | rs56751865 | T | 2.64E-12 | -12.79 |
| 11 | 116908706 | rs80273401 | C | 3.05E-12 | -14.29 |
| 11 | 116911012 | rs12273545 | T | 3.12E-12 | -14.29 |
| 11 | 116912117 | rs77999164 | T | 3.12E-12 | -14.29 |
| 11 | 116913570 | rs80143284 | A | 3.12E-12 | -14.29 |
| 11 | 116907715 | rs75216569 | G | 3.30E-12 | -14.27 |
| 11 | 116922586 | rs111530588 | A | 3.38E-12 | -14.27 |
| 11 | 116956990 | rs12281358 | C | 3.51E-12 | -14.26 |
| 11 | 116919273 | rs12280172 | A | 3.53E-12 | -14.25 |
| 11 | 116919969 | rs75890305 | G | 3.53E-12 | -14.25 |
| 11 | 116924000 | rs11216250 | T | 3.53E-12 | -14.25 |
| 11 | 116955934 | rs2368149 | A | 3.62E-12 | -14.25 |
| 11 | 116927762 | rs10892067 | C | 3.79E-12 | -14.23 |
| 11 | 116928546 | rs10892068 | T | 3.79E-12 | -14.23 |
| 11 | 116940210 | rs11216261 | C | 3.84E-12 | -14.23 |
| 11 | 116941540 | rs9971421 | T | 3.84E-12 | -14.23 |
| 11 | 116944846 | rs17120293 | C | 3.96E-12 | -14.22 |
| 11 | 116910971 | rs12273536 | T | 3.96E-12 | -14.20 |
| 11 | 116937361 | rs79955870 | T | 3.97E-12 | -14.22 |
| 11 | 116863047 | rs12272973 | C | 4.01E-12 | -14.16 |
| 11 | 116863458 | rs10128682 | T | 4.01E-12 | -14.16 |
| 11 | 116865100 | rs75826529 | A | 4.01E-12 | -14.16 |
| 11 | 116865207 | rs74460214 | C | 4.01E-12 | -14.16 |
| 11 | 116865367 | rs79349393 | G | 4.01E-12 | -14.16 |
| 11 | 116870411 | rs10892056 | C | 4.01E-12 | -14.16 |
| 11 | 116928223 | rs11216252 | A | 4.02E-12 | -14.21 |
| 11 | 116928225 | rs11216253 | C | 4.02E-12 | -14.21 |
| 11 | 116943066 | rs116582417 | A | 4.07E-12 | -14.21 |
| 11 | 116970761 | rs76567973 | G | 4.08E-12 | -14.82 |
| 11 | 116946853 | rs12274725 | T | 4.14E-12 | -14.20 |
| 11 | 116947180 | rs11216265 | C | 4.14E-12 | -14.20 |
| 11 | 116902372 | rs7481286 | T | 4.23E-12 | -14.21 |
| 11 | 116850351 | rs11216212 | A | 4.24E-12 | -14.15 |
| 11 | 116851367 | rs146178826 | G | 4.24E-12 | -14.15 |
| 11 | 116852687 | rs11216215 | A | 4.24E-12 | -14.15 |
| 11 | 116853922 | rs10128690 | A | 4.43E-12 | -14.14 |

| | | | | | |
|---|---|---|---|---|---|
| 11 | 116854021 | rs10128570 | G | 4.43E-12 | -14.14 |
| 11 | 116856322 | rs12287768 | C | 4.43E-12 | -14.14 |
| 11 | 116856698 | rs12274771 | G | 4.43E-12 | -14.14 |
| 11 | 116856709 | rs12274773 | T | 4.43E-12 | -14.14 |
| 11 | 116856992 | rs75212922 | A | 4.43E-12 | -14.14 |
| 11 | 116857652 | rs11216217 | A | 4.43E-12 | -14.14 |
| 11 | 116862245 | rs80242245 | G | 4.43E-12 | -14.14 |
| 11 | 116862661 | rs11216218 | T | 4.43E-12 | -14.14 |
| 11 | 116862755 | rs11216219 | T | 4.43E-12 | -14.14 |
| 11 | 116842676 | rs12418324 | T | 4.74E-12 | -14.12 |
| 11 | 116846266 | rs74927399 | C | 4.74E-12 | -14.12 |
| 11 | 116846343 | rs76949321 | A | 4.74E-12 | -14.12 |
| 11 | 116848186 | rs11216209 | C | 4.74E-12 | -14.12 |
| 11 | 116985191 | rs74882651 | A | 4.80E-12 | -14.25 |
| 11 | 116988790 | rs80197460 | G | 4.80E-12 | -14.19 |
| 11 | 116953644 | rs11216270 | A | 4.81E-12 | -14.17 |
| 11 | 116886721 | rs79870586 | A | 4.86E-12 | -14.17 |
| 11 | 116890667 | rs80054374 | G | 4.86E-12 | -14.17 |
| 11 | 116892368 | rs77955134 | A | 4.86E-12 | -14.17 |
| 11 | 116892446 | rs77594377 | A | 4.86E-12 | -14.17 |
| 11 | 116971677 | rs78135964 | T | 4.90E-12 | -14.77 |
| 11 | 116951584 | rs10082561 | C | 4.92E-12 | -14.16 |
| 11 | 116827369 | rs17120177 | C | 5.08E-12 | -14.14 |
| 11 | 116896913 | rs2186844 | T | 5.12E-12 | -14.15 |
| 11 | 116976009 | rs142395187 | G | 5.18E-12 | -14.52 |
| 11 | 116899580 | rs12289032 | T | 5.31E-12 | -14.14 |
| 11 | 116899825 | rs146943088 | C | 5.31E-12 | -14.14 |
| 11 | 116823107 | rs77467134 | C | 5.35E-12 | -14.08 |
| 11 | 116823521 | rs76654964 | A | 5.35E-12 | -14.08 |
| 11 | 116823617 | rs76146269 | G | 5.35E-12 | -14.08 |
| 11 | 116824162 | rs12275355 | G | 5.35E-12 | -14.08 |
| 11 | 116824286 | rs12289974 | T | 5.35E-12 | -14.08 |
| 11 | 116824620 | rs2289890 | A | 5.35E-12 | -14.08 |
| 11 | 116825240 | rs2289892 | A | 5.35E-12 | -14.08 |
| 11 | 116826617 | rs12291040 | C | 5.35E-12 | -14.08 |
| 11 | 116826643 | rs12270837 | C | 5.35E-12 | -14.08 |
| 11 | 116831016 | rs11216199 | T | 5.35E-12 | -14.08 |
| 11 | 116832718 | rs10892051 | A | 5.35E-12 | -14.08 |
| 11 | 116774244 | rs78694306 | T | 5.40E-12 | -15.42 |
| 11 | 116874358 | rs12278943 | C | 5.43E-12 | -14.07 |
| 11 | 116878066 | rs11216226 | G | 5.43E-12 | -14.07 |
| 11 | 116878759 | rs12276805 | C | 5.43E-12 | -14.07 |
| 11 | 116879723 | rs138181556 | A | 5.43E-12 | -14.07 |

| | | | | | |
|---|---|---|---|---|---|
| 11 | 116774499 | rs115971514 | C | 5.63E-12 | -15.36 |
| 11 | 116980679 | rs114221654 | T | 5.73E-12 | -14.21 |
| 11 | 116979363 | rs12416941 | A | 5.74E-12 | -14.21 |
| 11 | 116985857 | rs60834024 | A | 5.83E-12 | -14.18 |
| 11 | 116784692 | rs11216186 | C | 6.02E-12 | -14.05 |
| 11 | 116785341 | rs79711490 | T | 6.02E-12 | -14.05 |
| 11 | 116807173 | rs12273769 | G | 6.04E-12 | -14.04 |
| 11 | 116807368 | rs12294657 | C | 6.04E-12 | -14.04 |
| 11 | 116808453 | rs77136879 | T | 6.04E-12 | -14.04 |
| 11 | 116814646 | rs12421902 | G | 6.06E-12 | -14.04 |
| 11 | 116815599 | rs11216193 | T | 6.06E-12 | -14.04 |
| 11 | 116815738 | rs11216194 | C | 6.06E-12 | -14.04 |
| 11 | 116816394 | rs10892050 | A | 6.06E-12 | -14.04 |
| 11 | 116816778 | rs11216197 | G | 6.06E-12 | -14.04 |
| 11 | 116819264 | rs12293755 | T | 6.06E-12 | -14.04 |
| 11 | 116819906 | rs12419047 | A | 6.06E-12 | -14.04 |
| 11 | 116820023 | rs12421834 | C | 6.06E-12 | -14.04 |
| 11 | 116820690 | rs12419437 | A | 6.06E-12 | -14.04 |
| 11 | 116981515 | rs116770907 | T | 6.08E-12 | -14.19 |
| 11 | 116813438 | rs75410648 | T | 6.33E-12 | -14.03 |
| 11 | 116809789 | rs7480278 | G | 6.44E-12 | -14.02 |
| 11 | 116810906 | rs80015203 | G | 6.44E-12 | -14.02 |
| 11 | 116810954 | rs117877581 | G | 6.44E-12 | -14.02 |
| 11 | 116811487 | rs116597701 | G | 6.44E-12 | -14.02 |
| 11 | 116812058 | rs78066444 | C | 6.44E-12 | -14.02 |
| 11 | 116812628 | rs17120157 | C | 6.44E-12 | -14.02 |
| 11 | 116782974 | rs11216185 | G | 6.58E-12 | -15.31 |
| 11 | 116867514 | rs11604866 | T | 6.72E-12 | -14.02 |
| 11 | 116781545 | rs11216183 | A | 6.78E-12 | -15.33 |
| 11 | 116982434 | rs139960808 | T | 6.84E-12 | -14.17 |
| 11 | 116787327 | rs12277092 | T | 6.86E-12 | -14.02 |
| 11 | 116787633 | rs12418208 | T | 6.86E-12 | -14.02 |
| 11 | 116787641 | rs12418209 | T | 6.86E-12 | -14.02 |
| 11 | 116788133 | rs1006176 | C | 6.86E-12 | -14.02 |
| 11 | 116800270 | rs79157715 | A | 7.16E-12 | -14.00 |
| 11 | 116801061 | rs75776964 | G | 7.16E-12 | -14.00 |
| 11 | 116802972 | rs74615346 | C | 7.16E-12 | -14.00 |
| 11 | 116804005 | rs12270974 | T | 7.16E-12 | -14.00 |
| 11 | 116804370 | rs12272491 | T | 7.16E-12 | -14.00 |
| 11 | 116804762 | rs12418735 | G | 7.16E-12 | -14.00 |
| 11 | 116787320 | rs12277181 | A | 7.18E-12 | -13.99 |
| 11 | 116791307 | rs78692246 | A | 7.28E-12 | -14.00 |
| 11 | 116793693 | rs10892047 | T | 7.28E-12 | -14.00 |

| | | | | | |
|---|---|---|---|---|---|
| 11 | 116795948 | rs12292371 | C | 7.28E-12 | -14.00 |
| 11 | 116796065 | rs12292434 | A | 7.28E-12 | -14.00 |
| 11 | 116797475 | rs12285956 | A | 7.28E-12 | -14.00 |
| 11 | 116879679 | rs117901629 | A | 7.67E-12 | -14.05 |
| 11 | 116992685 | rs74955808 | A | 9.75E-12 | -13.88 |
| 11 | 116838130 | rs12281729 | C | 1.03E-11 | -13.84 |
| 11 | 116839293 | rs12417152 | C | 1.03E-11 | -13.84 |
| 11 | 116839614 | rs11216204 | A | 1.03E-11 | -13.84 |
| 11 | 116839938 | rs12272305 | A | 1.03E-11 | -13.84 |
| 11 | 116840033 | rs12279266 | C | 1.03E-11 | -13.84 |
| 11 | 116975136 | rs149576335 | A | 1.06E-11 | -14.35 |
| 11 | 116940449 | rs12280210 | C | 1.09E-11 | -12.08 |
| 11 | 116828939 | rs878692 | A | 1.11E-11 | -13.81 |
| 11 | 116829442 | rs12420857 | T | 1.11E-11 | -13.81 |
| 11 | 116822528 | rs12286405 | T | 1.17E-11 | -12.00 |
| 11 | 116973247 | rs76942203 | A | 1.18E-11 | -14.38 |
| 11 | 116792961 | rs7948838 | G | 1.25E-11 | -11.95 |
| 11 | 116797684 | rs11216190 | C | 1.27E-11 | -11.95 |
| 11 | 116816216 | rs10892049 | C | 1.28E-11 | -11.91 |
| 11 | 116820088 | rs12420518 | C | 1.28E-11 | -11.95 |
| 11 | 116774475 | rs78832854 | A | 1.29E-11 | -14.94 |
| 11 | 116863988 | rs11216220 | C | 1.33E-11 | -11.99 |
| 11 | 116866240 | rs7931665 | G | 1.33E-11 | -11.99 |
| 11 | 116851608 | rs7358350 | C | 1.37E-11 | -11.98 |
| 11 | 116848329 | rs11216210 | T | 1.43E-11 | -11.97 |
| 11 | 116596309 | rs61905078 | C | 1.51E-11 | -13.34 |
| 11 | 116875924 | rs11216224 | C | 1.54E-11 | -11.96 |
| 11 | 116831843 | rs11216200 | T | 1.54E-11 | -11.94 |
| 11 | 116998912 | rs145802214 | C | 1.57E-11 | -13.74 |
| 11 | 116707684 | rs2070665 | A | 1.58E-11 | 9.21 |
| 11 | 116707583 | rs5072 | A | 1.59E-11 | 9.21 |
| 11 | 116879224 | rs12271319 | T | 1.61E-11 | -11.94 |
| 11 | 117000179 | rs146067038 | G | 1.61E-11 | -13.76 |
| 11 | 116879519 | rs12284696 | G | 1.63E-11 | -11.93 |
| 11 | 116813979 | rs12292858 | C | 1.64E-11 | -11.82 |
| 11 | 116817655 | rs191655881 | C | 1.68E-11 | -11.87 |
| 11 | 116875152 | rs7946390 | G | 1.72E-11 | -11.92 |
| 11 | 116876442 | rs1940625 | G | 1.72E-11 | -11.92 |
| 11 | 116878014 | rs11216225 | G | 1.72E-11 | -11.92 |
| 11 | 116880456 | rs7112937 | C | 1.72E-11 | -11.92 |
| 11 | 116870480 | rs10502224 | G | 1.88E-11 | -11.86 |
| 11 | 117002828 | rs17120367 | T | 2.00E-11 | -13.69 |
| 11 | 117003388 | rs17120370 | C | 2.00E-11 | -13.69 |

| | | | | | |
|---|---|---|---|---|---|
| 11 | 117003418 | rs74735277 | T | 2.00E-11 | -13.69 |
| 11 | 117003623 | rs75208249 | C | 2.00E-11 | -13.69 |
| 11 | 116996345 | rs17120344 | A | 2.06E-11 | -13.65 |
| 11 | 116996711 | rs12419459 | T | 2.06E-11 | -13.64 |
| 11 | 116996631 | rs12419454 | T | 2.09E-11 | -13.64 |
| 11 | 116996783 | rs12416691 | T | 2.09E-11 | -13.64 |
| 11 | 117001960 | rs12421425 | T | 2.10E-11 | -13.68 |
| 11 | 116602399 | rs75387300 | A | 2.19E-11 | -13.24 |
| 11 | 116999025 | rs141250514 | C | 2.28E-11 | -13.63 |
| 11 | 116998306 | rs12420200 | A | 2.52E-11 | -13.60 |
| 11 | 116837089 | rs78044162 | T | 2.56E-11 | -11.82 |
| 11 | 116837373 | rs9971422 | C | 2.60E-11 | -11.78 |
| 11 | 116563992 | rs1787701 | C | 2.72E-11 | -9.34 |
| 11 | 116999600 | rs138039720 | T | 2.79E-11 | -13.57 |
| 11 | 116993788 | rs12420361 | A | 2.96E-11 | -13.55 |
| 11 | 116788837 | rs1135663 | T | 3.51E-11 | -13.66 |
| 11 | 117004853 | rs12417098 | A | 3.62E-11 | -13.50 |
| 11 | 117004201 | rs114945429 | T | 3.63E-11 | -13.50 |
| 11 | 116854181 | rs10128571 | G | 3.64E-11 | -13.59 |
| 11 | 116598065 | rs78484485 | A | 3.71E-11 | -10.88 |
| 11 | 117010091 | rs115991973 | A | 3.72E-11 | -13.46 |
| 11 | 117010123 | rs116384608 | G | 3.72E-11 | -13.46 |
| 11 | 117010474 | rs77661993 | C | 4.19E-11 | -13.43 |
| 11 | 116566763 | rs59854309 | T | 4.27E-11 | -10.04 |
| 11 | 116597889 | rs180363 | C | 4.60E-11 | -10.77 |
| 11 | 117019867 | rs139146464 | G | 4.79E-11 | -13.32 |
| 11 | 116815891 | rs11216195 | A | 4.83E-11 | -13.57 |
| 11 | 116815892 | rs11216196 | G | 4.83E-11 | -13.57 |
| 11 | 117074299 | rs2269397 | C | 4.84E-11 | -12.95 |
| 11 | 116577043 | rs1263055 | A | 4.95E-11 | -9.89 |
| 11 | 116818343 | rs146056222 | G | 5.41E-11 | -11.61 |
| 11 | 116596752 | rs180364 | G | 5.77E-11 | -10.74 |
| 11 | 116672231 | rs2542061 | G | 6.87E-11 | -8.96 |
| 11 | 116566933 | rs60954647 | T | 7.34E-11 | -8.28 |
| 11 | 117017455 | rs12419436 | G | 7.40E-11 | -13.23 |
| 11 | 117017530 | rs12420725 | G | 7.40E-11 | -13.23 |
| 11 | 117017714 | rs12417984 | A | 7.40E-11 | -13.23 |
| 11 | 116598988 | rs180360 | G | 7.95E-11 | -10.67 |
| 11 | 117016450 | rs2306471 | T | 8.68E-11 | -13.18 |
| 11 | 116594031 | rs12420920 | A | 9.10E-11 | -12.99 |
| 11 | 116594294 | rs12421191 | T | 9.10E-11 | -12.99 |
| 11 | 116593107 | rs79100692 | A | 9.32E-11 | -13.01 |
| 11 | 116592460 | rs74680844 | C | 9.47E-11 | -13.00 |

| | | | | | |
|---|---|---|---|---|---|
| 11 | 117023706 | rs143494314 | T | 9.59E-11 | -13.18 |
| 11 | 117025932 | rs12418349 | G | 9.59E-11 | -13.18 |
| 11 | 117028727 | rs12420250 | A | 1.06E-10 | -13.15 |
| 11 | 117015238 | rs12419227 | A | 1.08E-10 | -13.08 |
| 11 | 117029785 | rs78166985 | T | 1.09E-10 | -13.15 |
| 11 | 117085210 | rs77683187 | C | 1.38E-10 | -12.87 |
| 11 | 117031363 | rs142017584 | A | 1.40E-10 | -13.08 |
| 11 | 116552766 | rs1787717 | T | 1.70E-10 | -8.67 |
| 11 | 117080640 | rs11216315 | G | 1.71E-10 | -12.79 |
| 11 | 117082966 | rs76169968 | A | 1.75E-10 | -12.79 |
| 11 | 116701535 | rs4520 | C | 1.94E-10 | -8.58 |
| 11 | 117035016 | rs150984554 | A | 2.01E-10 | -12.88 |
| 11 | 117032959 | rs12281009 | G | 2.08E-10 | -12.94 |
| 11 | 116801201 | rs645258 | A | 2.08E-10 | 8.96 |
| 11 | 117005627 | rs141936239 | C | 2.15E-10 | -12.72 |
| 11 | 117015516 | rs12419272 | G | 2.27E-10 | -12.80 |
| 11 | 117084396 | rs12418705 | T | 3.03E-10 | -12.69 |
| 11 | 117050918 | rs12419178 | T | 3.25E-10 | -12.70 |
| 11 | 117038741 | rs77171490 | A | 3.27E-10 | -12.71 |
| 11 | 117039461 | rs116773440 | C | 3.27E-10 | -12.71 |
| 11 | 117040505 | rs77678768 | C | 3.27E-10 | -12.71 |
| 11 | 117048122 | rs80353869 | A | 3.27E-10 | -12.71 |
| 11 | 116591553 | rs180373 | A | 3.28E-10 | -12.65 |
| 11 | 117051725 | rs76883864 | T | 3.30E-10 | -12.71 |
| 11 | 117074109 | rs12970 | A | 3.36E-10 | -12.68 |
| 11 | 116570968 | rs1145194 | A | 3.53E-10 | -9.60 |
| 11 | 116671476 | rs6589569 | T | 3.65E-10 | 8.87 |
| 11 | 117069023 | rs7112573 | C | 3.87E-10 | -12.50 |
| 11 | 116564562 | rs1787698 | T | 3.93E-10 | -9.60 |
| 11 | 117056059 | rs11606672 | T | 3.96E-10 | -12.64 |
| 11 | 117054709 | rs2046149 | G | 4.00E-10 | -12.64 |
| 11 | 117035319 | rs12420127 | G | 4.03E-10 | -12.62 |
| 11 | 117053433 | rs2046150 | A | 4.05E-10 | -12.64 |
| 11 | 116564553 | rs1787699 | G | 4.10E-10 | -9.59 |
| 11 | 117082109 | rs12417682 | A | 4.23E-10 | -12.61 |
| 11 | 116565055 | rs1784118 | T | 4.24E-10 | -9.58 |
| 11 | 116858994 | rs10160754 | C | 4.38E-10 | -11.54 |
| 11 | 117058062 | rs2306472 | T | 4.40E-10 | -12.61 |
| 11 | 117059131 | rs188351692 | T | 4.47E-10 | -12.60 |
| 11 | 117059794 | rs7933134 | C | 4.67E-10 | -12.59 |
| 11 | 117061775 | rs12419470 | T | 4.67E-10 | -12.59 |
| 11 | 117068444 | rs74385934 | A | 4.85E-10 | -12.56 |
| 11 | 117064776 | rs11605883 | G | 4.86E-10 | -12.52 |

| | | | | | |
|---|---|---|---|---|---|
| 11 | 117065066 | rs3781985 | C | 4.86E-10 | -12.52 |
| 11 | 117065153 | rs80183569 | A | 4.86E-10 | -12.52 |
| 11 | 116758693 | rs12292278 | C | 4.90E-10 | -12.69 |
| 11 | 117070596 | rs2239678 | T | 5.00E-10 | -12.55 |
| 11 | 117063709 | rs2301174 | G | 5.07E-10 | -12.53 |
| 11 | 117065533 | rs11606480 | A | 5.10E-10 | -12.51 |
| 11 | 117065772 | rs112712475 | A | 5.10E-10 | -12.51 |
| 11 | 117070740 | rs2170317 | C | 5.15E-10 | -12.53 |
| 11 | 116574817 | rs1240781 | T | 5.22E-10 | -9.53 |
| 11 | 116562805 | rs1784119 | T | 5.46E-10 | -9.51 |
| 11 | 116753552 | rs12275565 | G | 5.48E-10 | -12.66 |
| 11 | 116750073 | rs543819 | G | 5.87E-10 | -12.65 |
| 11 | 116670676 | rs6589567 | A | 5.98E-10 | 8.68 |
| 11 | 116705719 | rs12718462 | C | 6.10E-10 | -15.63 |
| 11 | 116671823 | rs9667814 | C | 6.11E-10 | 8.77 |
| 11 | 116577441 | rs11216105 | A | 6.18E-10 | -9.20 |
| 11 | 116671824 | rs9666150 | T | 6.21E-10 | 8.76 |
| 11 | 116571138 | rs11601168 | A | 6.26E-10 | -15.14 |
| 11 | 116703640 | rs5128 | G | 6.30E-10 | 8.43 |
| 11 | 116759824 | rs12294191 | G | 6.36E-10 | -12.54 |
| 11 | 117062489 | rs3213459 | C | 6.36E-10 | -12.47 |
| 11 | 116569204 | rs947986 | A | 6.64E-10 | -9.46 |
| 11 | 116706047 | rs12721025 | A | 6.67E-10 | -15.60 |
| 11 | 116560967 | rs3017229 | A | 6.69E-10 | -9.47 |
| 11 | 116567449 | rs1784106 | A | 6.83E-10 | -9.46 |
| 11 | 116671005 | rs4938313 | A | 7.18E-10 | 8.65 |
| 11 | 116560610 | rs1787714 | A | 7.39E-10 | -9.43 |
| 11 | 116568516 | rs1787690 | T | 8.57E-10 | -9.39 |
| 11 | 116560611 | rs1787713 | G | 8.60E-10 | -9.40 |
| 11 | 117061825 | rs61703672 | T | 8.61E-10 | -12.40 |
| 11 | 117089722 | rs2239009 | C | 9.12E-10 | -12.30 |
| 11 | 116706168 | rs12721026 | G | 9.16E-10 | -15.65 |
| 11 | 116672013 | rs11216140 | T | 9.65E-10 | 8.71 |
| 11 | 117069788 | rs2269398 | T | 9.87E-10 | -12.39 |
| 11 | 116707401 | rs12718464 | A | 1.01E-09 | -15.61 |
| 11 | 117088082 | rs2238005 | T | 1.03E-09 | -12.27 |
| 11 | 117005751 | rs143261386 | C | 1.03E-09 | -12.12 |
| 11 | 116681563 | rs6589572 | G | 1.10E-09 | 8.36 |
| 11 | 117092051 | rs78628783 | T | 1.18E-09 | -12.22 |
| 11 | 117092394 | rs80351364 | T | 1.22E-09 | -12.21 |
| 11 | 116705516 | rs525028 | A | 1.26E-09 | -9.11 |
| 11 | 116921390 | rs1815786 | T | 1.50E-09 | 8.71 |
| 11 | 116682156 | rs7927820 | G | 1.55E-09 | 8.26 |

| | | | | | |
|---|---|---|---|---|---|
| 11 | 116707338 | rs7116797 | A | 1.59E-09 | 8.04 |
| 11 | 116957907 | rs6589592 | C | 1.68E-09 | 8.47 |
| 11 | 116809222 | rs187184721 | T | 1.68E-09 | -15.50 |
| 11 | 116684028 | rs7396835 | T | 1.76E-09 | 8.21 |
| 11 | 116684164 | rs7396851 | T | 1.76E-09 | 8.21 |
| 11 | 116765925 | rs74881320 | C | 1.77E-09 | -12.31 |
| 11 | 116908283 | rs7115242 | A | 1.92E-09 | 8.44 |
| 11 | 116588425 | rs4938304 | T | 2.01E-09 | -15.02 |
| 11 | 116765476 | rs77349713 | C | 2.03E-09 | -12.26 |
| 11 | 116778813 | rs35184536 | G | 2.23E-09 | -12.30 |
| 11 | 116772486 | rs17120131 | C | 2.23E-09 | -12.24 |
| 11 | 116773318 | rs17120132 | A | 2.30E-09 | -12.24 |
| 11 | 116862818 | rs607273 | A | 2.58E-09 | 8.36 |
| 11 | 116773761 | rs17120136 | G | 2.70E-09 | -12.22 |
| 11 | 116843425 | rs11216206 | G | 2.80E-09 | -8.03 |
| 11 | 117092886 | rs6589606 | T | 2.96E-09 | -11.99 |
| 11 | 116702123 | rs5141 | T | 3.02E-09 | 8.14 |
| 11 | 116777063 | rs12273762 | A | 3.03E-09 | -12.16 |
| 11 | 116775196 | rs12280030 | A | 3.04E-09 | -12.15 |
| 11 | 116685766 | rs6589573 | C | 3.04E-09 | 8.09 |
| 11 | 116778065 | rs9651681 | C | 3.26E-09 | -12.13 |
| 11 | 116705568 | rs10750098 | G | 3.45E-09 | 8.06 |
| 11 | 116510558 | rs184616707 | G | 4.13E-09 | 34.11 |
| 11 | 116515405 | rs11216071 | C | 4.31E-09 | 11.58 |
| 11 | 116526322 | rs486394 | C | 4.50E-09 | 11.19 |
| 11 | 116679155 | rs75542613 | A | 5.00E-09 | -16.21 |
| 11 | 116532548 | rs118175510 | C | 5.22E-09 | 12.71 |
| 11 | 116538576 | rs1784127 | A | 5.43E-09 | -7.84 |
| 11 | 116538689 | rs1784128 | A | 5.43E-09 | -7.84 |
| 11 | 116565690 | rs116978945 | T | 6.07E-09 | 11.64 |
| 11 | 116675294 | rs6589570 | A | 6.59E-09 | 9.19 |
| 11 | 116555415 | rs3016357 | T | 8.48E-09 | -8.48 |
| 11 | 116567613 | rs78888567 | A | 9.22E-09 | 11.46 |
| 11 | 116519181 | rs76234156 | A | 9.29E-09 | 11.14 |
| 11 | 116555422 | rs1145213 | G | 1.05E-08 | -8.42 |
| 11 | 116538215 | rs1784126 | T | 1.05E-08 | -7.68 |
| 11 | 116536281 | rs1240776 | T | 1.26E-08 | -7.63 |
| 11 | 116611995 | rs180348 | A | 1.82E-08 | -14.24 |
| 11 | 116839761 | rs1241657 | T | 1.88E-08 | 7.88 |
| 11 | 116557216 | rs1145211 | A | 2.09E-08 | -8.25 |
| 11 | 116686288 | rs7107757 | T | 2.51E-08 | 7.27 |
| 11 | 116701850 | rs5142 | T | 2.74E-08 | 7.61 |
| 11 | 116680418 | rs6589571 | T | 3.15E-08 | 8.75 |

| | | | | | |
|---|---|---|---|---|---|
| 11 | 116692449 | rs6413456 | A | 3.75E-08 | -15.61 |
| 11 | 116545723 | rs1787688 | C | 3.78E-08 | -8.05 |
| 11 | 116565309 | rs12293222 | A | 3.89E-08 | 7.82 |

**S3 Table.** Residual hydrogen bond analysis. The details of different H-bonds in APOA5-WT and APOA5-MUT systems, along with H-bond donor and acceptor, the donor-acceptor distance (D-A distance) and the distance between H and acceptor has been given.

| | **Donor** | **Acceptor** | **Hydrogen** | **D-A distance (Å)** | **DH-A distance (Å)** |
|---|---|---|---|---|---|
| **APOA5-WT** | R 84.A NH1 | D 88.A OD1 | R 84.A 2HH1 | 3.415 | 2.652 |
| | Q 97.A NE2 | Q 329.B OE1 | Q 97.A 2HE2 | 2.942 | 2.163 |
| | H 205.A NE2 | T 307.A OG1 | H 205.A HE2 | 2.884 | 1.959 |
| | Q 127.B NE2 | E 123.B OE1 | Q 127.B 1HE2 | 2.957 | 2.159 |
| | T 133.B OG1 | T 300.A OG1 | T 133.B HG1 | 3.276 | 2.352 |
| | R 179.B NH1 | H 182.B ND1 | R 179.B 1HH1 | 3.145 | 2.298 |
| | Q 286.B NE2 | Q 290.B OE1 | Q 286.B 2HE2 | 3.461 | 2.509 |
| | Q 312.B NE2 | E 308.B OE2 | Q 312.B 1HE2 | 3.159 | 2.231 |
| **APOA5-MUT** | R 93.A NE | Q 329.B OE1 | R 93.A HE | 2.924 | 2.077 |
| | Q 139.A NE2 | D 135.A OD2 | Q 139.A 2HE2 | 3.041 | 2.195 |
| | H 210.A NE2 | S 225.A OG | H 210.A HE2 | 3.239 | 2.255 |
| | H 243.A NE2 | Q 247.A OE1 | H 243.A HE2 | 2.649 | 1.694 |
| | R 254.A NH1 | T 184.A OG1 | R 254.A 1HH1 | 3.143 | 2.148 |
| | T 266.A OG1 | E 268.B OE1 | T 266.A HG1 | 2.62 | 1.668 |
| | S 333.A OG | E 101.B OE2 | S 333.A HG1 | 2.674 | 1.757 |
| | K 130.B NZ | N 127.B OE1 | K 130.B HZ3 | 2.77 | 1.801 |
| | H 192.B NE2 | E 196.B OE2 | H 192.B HE2 | 3.007 | 2.076 |
| | N 249.B ND2 | T 292.B OG1 | N 249.B 1HD2 | 2.939 | 1.948 |
| | R 254.B NH1 | T 184.B OG1 | R 254.B 1HH1 | 2.974 | 2.077 |
| | Q 305.B NE2 | E 308.B OE1 | Q 305.B 2HE2 | 3.335 | 2.462 |
| | Q 313.B NE2 | Q 97.A OE1 | Q 313.B 2HE2 | 3.014 | 2.021 |
| | Q 330.B NE2 | E 327.B OE1 | Q 330.B 1HE2 | 2.862 | 1.865 |
